# A preclinical study of a novel dual-modality contrast agent in rodent models

**DOI:** 10.3389/fbioe.2025.1557772

**Published:** 2025-03-20

**Authors:** Xuelai Zhou, Kangli Jiang, Yuxin Han, Shuxu Yang

**Affiliations:** ^1^ Zhejiang Poly Pharm. Co., Ltd., Hangzhou, China; ^2^ Department of Neurosurgery, Sir Run Run Shaw Hospital, College of Medicine, Zhejiang University, Hangzhou, China

**Keywords:** gadolinium contrast agent, ICG, magnetic resonance imaging, fluorescence imaging, glioblastoma, intraoperative navigation

## Abstract

**Introduction:**

Glioblastoma (GBM) represents the most aggressive and prevalent form of primary malignant brain tumor in adults, with surgical intervention being the primary treatment modality. To enhance surgical outcomes and extend patient survival, we have engineered a dual-modality MRI/FI contrast agent known as PL002 to aid in the surgical management of GBM.

**Methods:**

In this study, an orthotopic glioma model was established in mice via intracranial injection of U-87 MG cells. Subsequently, the model animals were intravenously injected with PL002 and placed in a 7.0T magnetic resonance imaging (MRI) device to evaluate the imaging effects. After the MRI scan, fluorescence imaging techniques were employed to observe the distribution of PL002 at both the brain tissue and cellular levels. Moreover, healthy rat models were utilized to investigate the pharmacokinetic characteristics, tissue distribution, and safety profile of PL002.

**Results:**

The molecular structure of PL002 contains both gadolinium (Gd^3+^) and indocyanine green (ICG), demonstrating optimal imaging effects within the dosage range of 10-50 mg/kg, with a half-life of 2.51 to 4.87 hours. Even at relatively low concentrations in the brain, PL002 can provide stable and sustained support for MRI and fluorescence imaging for up to 72 hours. No abnormalities were observed in rats at a dosage of 100 mg/kg.

**Discussion:**

Compared to Gadavist® and ICG, PL002 provided sustained support for MRI and FI of GBM for 72 h, with a broad therapeutic window. This dual-modality contrast agent holds significant potential and promise for applications in preoperative assessment of resection margins, real-time intraoperative guidance, and postoperative verification of the extent of resection.

## 1 Introduction

Glioblastoma (GBM) is the most aggressive malignant brain tumor in adults, constituting approximately 50.9% of all malignant brain tumors according to the 2016–2020 CBTRUS Statistical Report (Wirtz et al., 2000; Qi et al., 2024). Compared to other malignancies, GBM is characterized by a poor prognosis and a short median survival time (Ostrom et al., 2023). Until to now, surgery remains the standard approach for GBM management. The extent of tumor resection is positively correlated with progression-free survival and quality of life. To achieve the maximum safe resection, neurosurgeons often utilize additional tools such as neuronavigation, intraoperative magnetic resonance imaging (iMRI), and intraoperative fluorescence navigation (Wirtz et al., 2000; Staartjes et al., 2021; Schupper et al., 2021).

Neuronavigation relies on preoperative MRI to accurately pinpoint the lesion, select the optimal surgical approach, and reduce postoperative complications. However, it lacks the capability to offer real-time evaluation of tumor margins throughout the surgical procedure, with the exception of iMRI (Krivosheya et al., 2021; Li et al., 2017). But iMRI is time-consuming, costly, and does not significantly impact prognosis (Bonosi et al., 2023; Mosteiro et al., 2022). Intraoperative fluorescence navigation enhances the visualization of brain tumor tissue through selective fluorescence in tumor cells (Schupper et al., 2022), such as with 5-aminolevulinic acid (5-ALA) fluorescence-guided surgery, which has been shown to improve 6-month progression-free survival rates in randomized trials. However, the cost of 5-ALA and the need for specialized equipment limit its widespread use. Indocyanine green (ICG) is another compound used in intraoperative fluorescence navigation and can also be used for direct glioblastoma imaging, potentially improving the extent of resection (Acerbi et al., 2018). Clinically, each intraoperative imaging technology has its advantages and disadvantages, and no single imaging technology can address all pre- and post-surgical detection and diagnostic needs. The combination of multiple imaging modalities for intraoperative tumor recognition is gaining attention among neurosurgeons due to the limitations of single imaging modes. Studies (Eyüpoglu et al., 2012; Tsugu et al., 2011) have shown that the combination of intraoperative MRI and fluorescence guidance has a synergistic effect in glioma surgery, with the combination approach significantly improving the extent of tumor resection compared to fluorescence-guided surgery alone.

Given the clinical benefits of multimodal imaging, there is a need for contrast agents capable of both MRI and fluorescence enhancement. Our group has developed a compound that incorporates Gd^3+^ and ICG molecules, aiming to provide dual MRI and fluorescence imaging capabilities. This dual-modality agent is expected to offer complementary bioimaging information for GBM surgery, improving surgical outcomes and extending postoperative patient survival.

## 2 Materials and methods

### 2.1 Material

*tert*-butyl (10-aminodecy)carbamate (Nanjing Weichuangyuan Medical Technology Co., LTD.), Indocyanine green (Dandong medical innovation Pharmaceutical Co., LTD., 21081512), Gadavist^®^(Bayer, KT04KPK), PL002 (Zhejiang Poly Pharmaceutical Co., LTD., 00-003958-25-01), Cell Counting Kit-8(Beyotime Biotechnology). Other chemical reagents all came from Sahn Chemical Technology (Shanghai) Co., LTD.

L-O2(BOHUI Biotechnology (GUANGZHOU) Co., LTD., BH-C073), HEK293(National Collection of Authenticated Cell Culture, SCSP-5209), U-87 MG (Zhejiang Meisen Cell Technology Co., LTD., CTCC-001-0023), Culture medium and fetal bovine serum were purchased from Gibco Laboratories (Grand Island, NY), BALB/c nude mice (Zhejiang Weitong Lihua Experimental Animal Technology Co., LTD.), Sprague-Dawley rat (Beijing Vitonglihua Experimental Animal Technology Co., LTD.). All animal experiments were conducted with the approval of the Experimental Animal Welfare and Ethics Committee of Zhejiang Longchuan Biomedical Technology Co., LTD.

### 2.2 Synthesis

#### 2.2.1 PL002-SMA

N, N-dimethylformamide, cyclotenine, and sodium acetate were mixed in a reaction flask, then tert-butyl bromoacetate and more N, N-dimethylformamide were added. The reaction was maintained at 25°C for 17 h with pH between 7 and 8, yielding SMA-1 as a white solid after filtration. In a new vessel, potassium carbonate, acetonitrile, methyl bromoacetate, and SMA-1 were stirred at 25°C for 6 h, resulting in SMA-2 as a yellow oil after filtration and concentration. Tetrahydrofuran, SMA-2, sodium hydroxide, and water were mixed and stirred at 30°C for 4 h, then the pH was adjusted to 6 with hydrochloric acid. After partitioning and drying, PL002-SMA was obtained as a white solid.

#### 2.2.2 PL002-SMC

1,1,2-trimethyl-1H-benzo [e]indole, 1,4-butanolide, and toluene were mixed and refluxed at 110°C for 20–21 h to yield SMC-1 as a light blue solid after cooling and drying. In a new vessel, N, N-dimethylformamide was mixed with phosphorus oxychloride at 0°C–5°C, then cyclohexanone was added at 15°C–20°C, resulting in SMC-2 as an orange solid after filtration and drying. Toluene, SMC-1, SMC-2, and sulfolane were mixed, refluxed at 116°C–118°C for 5–6 h, and SMC-3 was obtained as a reddish-brown solid. Acetonitrile and p-hydroxyphenylpropionic acid were mixed with sodium hydroxide solution at 0°C–5°C, reacted at 25°C–30°C for 1 h, and SMC-4 was obtained as a gray solid. In another flask, DMSO and SMC-3 were mixed at 22°C, then SMC-4 was added, and after stirring, PL002-SMC was obtained as a dark green solid.

#### 2.2.3 PL002

N, N-dimethylformamide, N, N-diisopropylethylamine, PL002-SMA, and PL002-SMB were combined in a reaction flask. PyBOP and more N, N-dimethylformamide were added and the mixture reacted for 4–6 h, then filtered to get PL001-01 filtrate. Hydrobromoacetic acid and triethylsilane were added to the filtrate at 0°C, mixed, and reacted at 20°C for 5 h to yield PL002-02 solid. PL002-02 was dissolved in water, gadolinium oxide was added, and the mixture was reacted at 100°C for 1 h. The pH was adjusted with sodium hydroxide solution, and the salt was removed by nanofiltration. PL002-03 was obtained by freeze-drying. In a new vessel, dimethyl sulfoxide, N, N-dimethylformamide, and N,N-diisopropylethylamine were added, followed by PL002-SMC, 1H-benzotriazole-1-hydroxy-tripyrroalkyl hexafluorphosphate, and PL002-03 at 15°C, and reacted for 2 h. After filtration and drying, PL002-05 was obtained. The liquid was purified using a DAC400 system, and PL002 was obtained by lyophilization. It was then packaged in double-layer pharmaceutical low-density polyethylene bags and aluminum foil. The synthesis was completed using ChemDraw 21.

### 2.3 *In vitro* relaxivity

PL002 was prepared in six different concentrations (0.2, 0.5, 1.0, 1.5, 2.0, and 2.5 μg/mL) using ultrapure water and sequentially tested in the MZsoMR23-60H-I nuclear magnetic resonance (NMR) analyzer. The T1 acquisition was performed using an inversion recovery (IR) sequence with the following intrinsic parameters: SF = 12 MHz, O1 = 557,454.41 Hz, P1 = 5.8 μs, P2 = 9.6 μs. The adjustable parameters were set as follows: SW = 100 kHz, TW = 32,000 ms, RFD = 0.08 ms, RG1 = 20, NS = 4, DR = 1, PRG = 0, TE = 1 ms.

### 2.4 Cell proliferation assay

L-O2, HEK293, and U-87 MG cells were seeded in a 96-well plate at 2 × 10^5^ cells/well and incubated overnight before being treated with various concentrations of PL002. After 24 h, cell proliferation was assessed using the CCK-8 assay. The culture medium was replaced with 100 μL of CCK-8 solution (10% reagent, 90% medium) per well, and the plate was incubated for 45 min at 37°C. Absorbance at 450 nm was measured using a SpectraMax_iD5 ELISA reader.

### 2.5 Animal modal

Human glioblastoma U87-MG cells were cultured in flasks with a 75 cm^2^ base area, using standard methods and DMEM medium with 10% FBS and 1% P/S, under 37°C and 5% CO2. Anesthetized animals were placed on a stereotaxic frame, and a scalp incision was made. A burr hole was drilled in the skull without penetrating the dura mater. A 10 μL cell suspension with 1 × 10^6^ cells was injected to a depth of 3 mm, then slowly infused over 5 min. The needle was left for 5 more minutes before withdrawal, and the hole was sealed with bone wax. The incision was closed and animals were monitored daily for health, including behavior, appetite, activity, and weight, after recovery on a heating pad.

### 2.6 MR imaging and fluorescent imaging *in vivo*

For *in vivo* MR and fluorescent imaging, 24 male BALB/c nude mice weighing about 20 g were divided into five groups: PL002 groups with doses of 5, 10, 50 mg/kg, a Gadavist^®^ group with 18.14 mg/kg, and an ICG group with 5 mg/kg. Except for the high-dose PL002 and Gadavist^®^ groups with 3 mice each, other groups had 6 mice. Mice were anesthetized and positioned for pre-injection MR imaging and at various post-injection time points using the BRUKER 7.0T MRI Biospoin GmbH nuclear magnetic resonance imaging system. T1-weighted imaging was conducted at several time points, excluding the high-dose groups from the 24-h scan. Scan parameters included: slice thickness (SL) = 0.700 mm, repetition time (TR) = 1,100.00 ms, echo time (TE) = 0.00 ms, filed of view (FOV) = 20 × 20 cm^2^, image size = 192 × 192, bandwidth (BW) = 372.02 Hz, flip angle (FA) = 180.00. Mice from the 6 and 24-h MR Imaging groups were euthanized, and 2 from each group were selected for *ex vivo* fluorescence imaging at 745 nm excitation and 800 nm emission. The remaining mice’s brains were fixed, embedded, sectioned, and stained with Hematoxylin and Eosin for histological examination. The same procedure was applied to the ICG group post-administration at 6 and 24 h.

All the animals were housed in a specific pathogen-free (SPF) animal facility and were removed from the facility on the day of imaging for the experiments.

### 2.7 Image analysis

After acquiring the MRI imaging data, manually trace the regions of interest (ROIs) for the tumor and normal tissues using ParaVision 360 software, and measure the signal intensity within these ROIs. Calculate the contrast-to-noise ratio (CNR) with the following formulas:

$$CNR=\frac{\left(S_{1}-S_{2}\right)}{SD}$$

where S_1_ is the signal intensity of the lesion, and S_2_ is the signal intensity of the normal tissue. Signal acquisition in the tumor region involves first manually identifying the tumor contour based on the image, followed by delineating the signal measurement area using software and recording the average signal intensity within this region. Signal acquisition in the normal tissue region is performed by selecting an area outside the tumor contour, delineating the measurement area using software, and recording the average signal intensity within this region. Signal acquisition in the background region involves selecting an area outside the brain tissue, delineating the measurement area using software, and recording the average signal intensity within this region.

Following the completion of fluorescence imaging, manually delineate the tumor region of interest (ROI) using the IVIS Spectrum software on the captured images, and measure the signal intensity. Calculate the mean fluorescence intensity contrast (CAR) with the following formula:

$$CAR=\frac{F_{1}}{F_{2}}$$

where F_1_ is the mean fluorescence intensity of the tumor, and F_2_ is the mean fluorescence intensity of the normal tissue. For fluorescence acquisition in the tumor region, the tumor contour was first manually identified by integrating the fluorescence image with the original photograph. Subsequently, the signal measurement area was delineated using software, and the average signal intensity within this region was recorded. For signal acquisition in the normal tissue region, an area outside the tumor contour was selected. The measurement area was then delineated using software, and the average signal intensity within this region was recorded.

### 2.8 HE staining

After fixation in a 4% paraformaldehyde solution, mouse brains are embedded in paraffin with a HistoStar machine (Thermo) and sectioned to about 3 μm thickness using an HM 340E slicer (Thermo). The sections are deparaffinized with xylene and stained with hematoxylin and eosin. Post-staining, they are dehydrated through ethanol solutions, cleared with xylene, and mounted with neutral balsam. Light microscopy is used to observe tumor cell necrosis, number, density, and morphological changes.

### 2.9 *In vivo* pharmacokinetics, tissue distribution and safety tests

In the pharmacokinetic study, Sprague-Dawley rats (n = 18, 3/sex/group) were administered the test article PL002 via intravenous infusion at doses of 3, 10, and 30 mg/kg, with an infusion duration of approximately 4 min. Blood samples were collected from all animals at various time points: pre-dose, 4 min (immediately after infusion), 10, 15, 30, and 45 min, and 1, 2, 4, 6, 10, 24, and 48 h post-administration. Heparin sodium was utilized as an anticoagulant in this study. After blood sample collection, the samples were transferred to centrifuge tubes and manually mixed. Centrifugation was completed within 2 h. The centrifugation conditions were as follows: 3,000 g, at 2°C–8°C, for 10 min. After centrifugation, the plasma was collected into new centrifuge tubes and stored at below −60°C until analysis. LC-MS/MS (Applied Biosystems, 6500QTRAP) condition: The liquid chromatography (LC) system consists of a binary pump, an online degassing unit, an autosampler, and a column oven. The mass spectrometry (MS) system includes an electrospray ionization (ESI) source and a triple-quadrupole mass spectrometer, which are used for LC and MS data acquisition as well as for chromatographic analysis. Chromatographic separation was performed at an injection temperature of 4°C using an ACE3 C4 analytical column (50 mm × 2.1 mm, 3 μm, ACE). The retention times for PL002 and glimepiride (internal standard) were 2.6 min and 2.5 min, respectively. The mobile phase consisted of A (0.1% formic acid in water) and B (0.1% formic acid in acetonitrile). Sample preparation: Take 50 μL of plasma sample, then add 150 μL of internal standard solution (20 ng/mL) and vortex mix. Centrifuge for 15 min at 4°C and 3,200 g. Take 100 μL of the supernatant, add 100 μL of acetonitrile, vortex mix, and then inject for analysis.

In the tissue distribution study, Sprague-Dawley rats (n = 24, 3/sex/group) were given PL002 at a dose of 10 mg/kg via intravenous infusion for about 4 min. Blood and tissue samples were harvested from the animals at 2, 6, 24, and 72 h after dosing. The tissues sampled included the heart, liver, spleen, lung, kidney, stomach, small intestine, large intestine (colon), gonads (epididymis/testis/ovary), brain, fat, muscle, and skin. Heparin sodium was used as an anticoagulant for blood samples. Blood samples were processed in the same manner as in the pharmacokinetic study after collection. Tissue samples were weighed, homogenized, and the homogenates were stored at −60°C or below. The analytical method for PL002 in blood is the same as that in the pharmacokinetic study. The analytical method for PL002 in tissues has been adjusted as follows: Chromatographic separation is performed with retention times of 2.4 min for PL002 and 2.5 min for glimepiride (internal standard). The mobile phase consists of A (0.2% formic acid in water) and B (0.2% formic acid in acetonitrile). Preprocessing of tissue homogenate samples: Take 50 μL of tissue homogenate sample, then add 150 μL of internal standard working solution (20 ng/mL, glimepiride), and vortex mix. Centrifuge for 15 min at 2°C–8°C and 3,200 g. Take 20 μL of the supernatant, add 180 μL of diluent (acetonitrile:water = 90:10, v/v), vortex mix, and then analyze by LC-MS/MS. The gadolinium content of plasma and tissue was measured by ICP-MS (Agilent, 7900 ICP-MS). The ICP-MS was operated in the standard instrument mode. Instrument control, data acquisition, and analysis were performed using Plasma Lab software. The instrument and operating conditions were optimized using a tuning solution. During the detection process, rhodium (Rh) was used as an internal standard for monitoring. Plasma Preprocessing Method: Take 50 μL of plasma sample, then add 10 μL of internal standard working solution (2 μg/mL, rhodium element) and vortex mix. Add 1 mL of diluent (65% nitric acid and Triton X-100, both with a volume fraction of 0.1%) to the above sample and vortex mix. Take 0.25 mL of the solution, add 4.0 mL of diluent, vortex mix, and then analyze by ICP-MS. Tissue Homogenate Preprocessing Method:** Take 100 μL of tissue homogenate sample, then add 10 μL of internal standard working solution (200 ng/mL, rhodium element) and vortex mix. Add 500 μL of digestion solution (65% nitric acid) to the above sample and vortex mix. Digest in a water bath (75°C) for 2 h. Take 400 μL of the digested solution, add 2.0 mL of diluent (65% nitric acid and Triton X-100, both with a volume fraction of 0.1%), vortex mix, and then analyze by ICP-MS.

In the toxicity study, Sprague-Dawley rats (n = 40, 5/sex/group) were treated with either vehicle control (5% Glucose Injection) or PL002 at doses of 100, 300, and 1,000 mg/kg via intravenous infusion, with a volume of 20 mL/kg and an infusion rate of 5 mL/kg/min. Throughout the study, mortality and morbidity, clinical signs, body weights, and food consumption were monitored. Gross observations were conducted on deceased animals and those euthanized on Day 15. The brains, hearts, livers, spleens, lungs, and kidneys of deceased animals, as well as tissues with gross lesions from all animals, were processed using standard histological techniques: paraffin embedding, sectioning, mounting on glass slides, and staining with hematoxylin and eosin (H&E). All slides were examined microscopically. The non-compartmental analysis (NCA) method with WinNonlin 8.0 software was employed to calculate the pharmacokinetic parameters of the administered group.

All the animals were housed in a specific pathogen-free (SPF) animal facility.

### 2.10 Data analysis and image processing

Statistical data are showed as mean ± standard deviation (SD). The CNR data from *in vivo* MRI at 6 h and the safety experiment data (analyzed separately for males and females) were analyzed using SPSS 22.0. Levene’s test was employed to assess the homogeneity of variance among groups. If the result of Levene’s test showed no significant difference (p ≥ 0.01), one-way analysis of variance (ANOVA) was used for variance (mean square error) analysis, and Dunnett’s test was applied for comparisons between the treatment groups and the control group. If Levene’s test showed a significant difference (p < 0.01), Welch’s test followed by Bonferroni correction was used for comparisons between the treatment groups and the control group. A two-tailed test was used with a confidence interval of 95%. P > 0.05 (NS) denotes there is no statistically significant. P < 0.05, p < 0.01, p < 0.001, were defined as statistically significant.

All data were processed and presented as images using GraphPad Prism 7 and Inkscape 1.4.

## 3 Results

### 3.1 Synthesis and characterization of PL002

**FIGURE 1:**
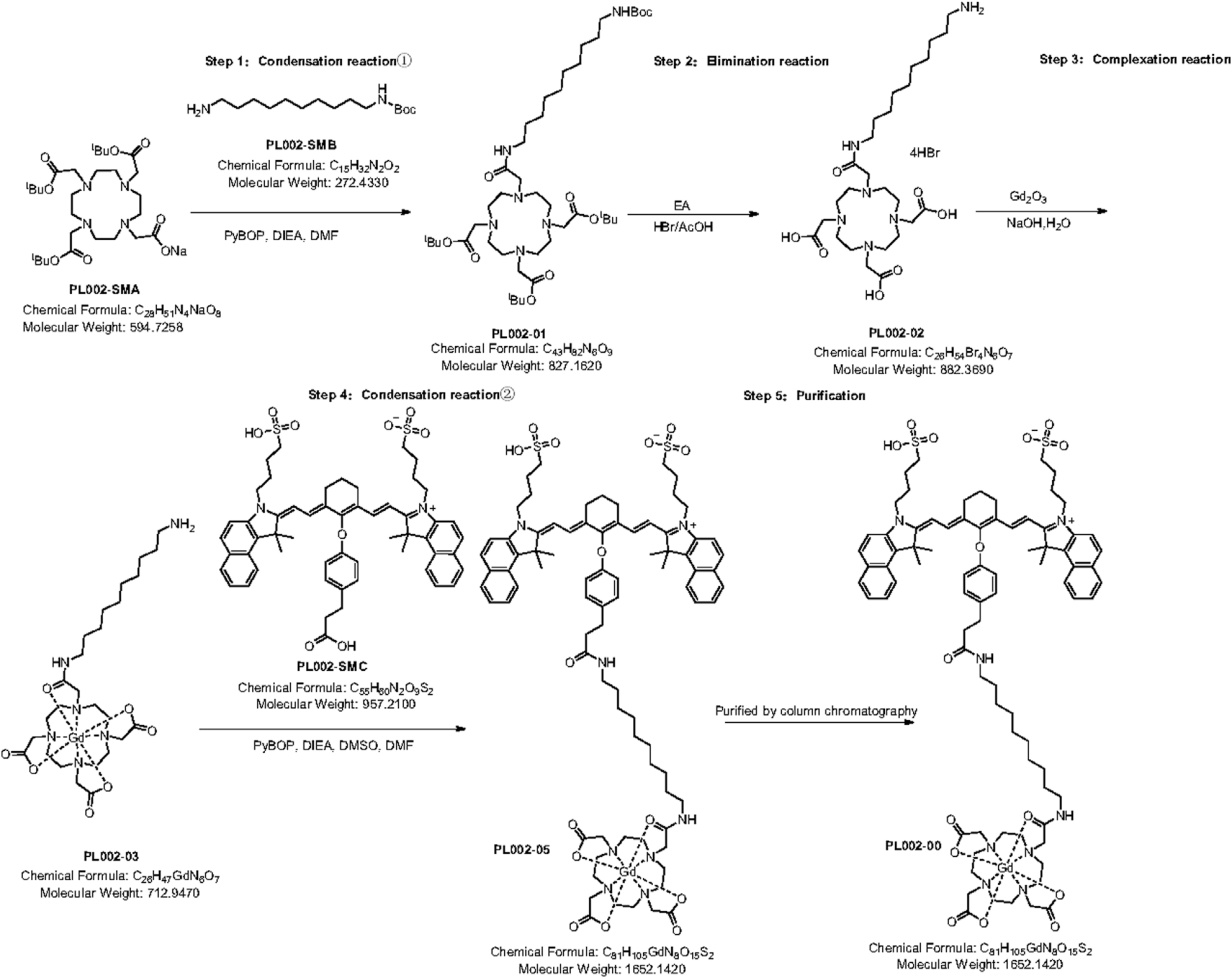
Synthetic route to PL002.

PL002 was synthesized utilizing PL002-SMA and PL002-SMB as the initial reactants, which underwent a condensation reaction (①), an elimination reaction, a coordination reaction, and a subsequent condensation reaction (②) with the starting material PL002-SMC to yield the final PL002 product. The synthetic route of PL002 is shown in Figure 1.

**FIGURE 2:**
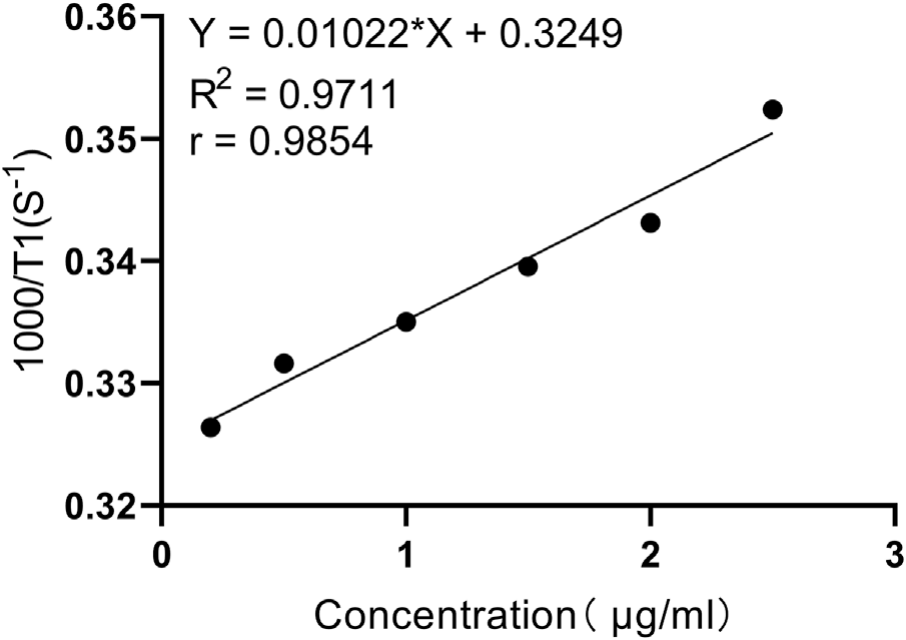
*In vitro* relaxation test curve (T1 sampling sequence: Intrinsic parameters SF = 12MHz, O1 = 557454.41Hz, P1 = 5.8us; Adjusted parameters SW = 1000KHz, TW = 32000 ms, RFD = 0.08 ms, RG1 = 20, DRG1 = 3, NS = 4, DR = 1, PRG = 0, TE = 1 ms.).

The UV spectrum indicates that the absorption band attributed to the benzene ring appears at the maximum wavelength λmax = 216.5 nm, while the absorption band due to Gd^3+^ is observed at the maximum wavelength λmax = 805.0 nm. These characteristics are in alignment with the structural formula of PL002. In the IR spectrum, the C-H stretching vibration absorption peak is detected at 2,922.86 cm^−1^, the C=O stretching vibration absorption peak for amide and carboxylic acid is found at 1,620.75 cm^−1^, and the peaks at 1,357.47 cm^−1^ and 1,115.75 cm^−1^ correspond to the SO2 stretching vibration absorption of the sulfonic acid group. The absorption peak at 1,039.05 cm^−1^ is associated with the C-N stretching vibration, all of which are consistent with the structural formula of PL002. High-resolution mass spectrometry (HRMS) captured the excimer ion peaks at m/z 1,652.6564 [M + H]^+^ and 826.8281 [M+2H]^2+^. The exact molecular weight measured was in close agreement with the theoretical calculation of 1,652.6460 for the predicted molecular formula C_81_H_106_N_8_O_15_S_2_Gd [M + H]^+^, with a difference (Diff) of 6.3 PPM, confirming the structural formula of PL002. Additionally, the relaxation rate test curve indicates that within the concentration range of 0–2.5ug/mL (0–1.5 μM in aqueous solution), as the concentration increases, the T1 relaxation time shortens and the relaxation rate enhances, suggesting that PL002 can serve as a T1-enhancing contrast agent for MRI (Figure 2).

### 3.2 MR imaging and fluorescent imaging *in vivo*

**FIGURE 3:**
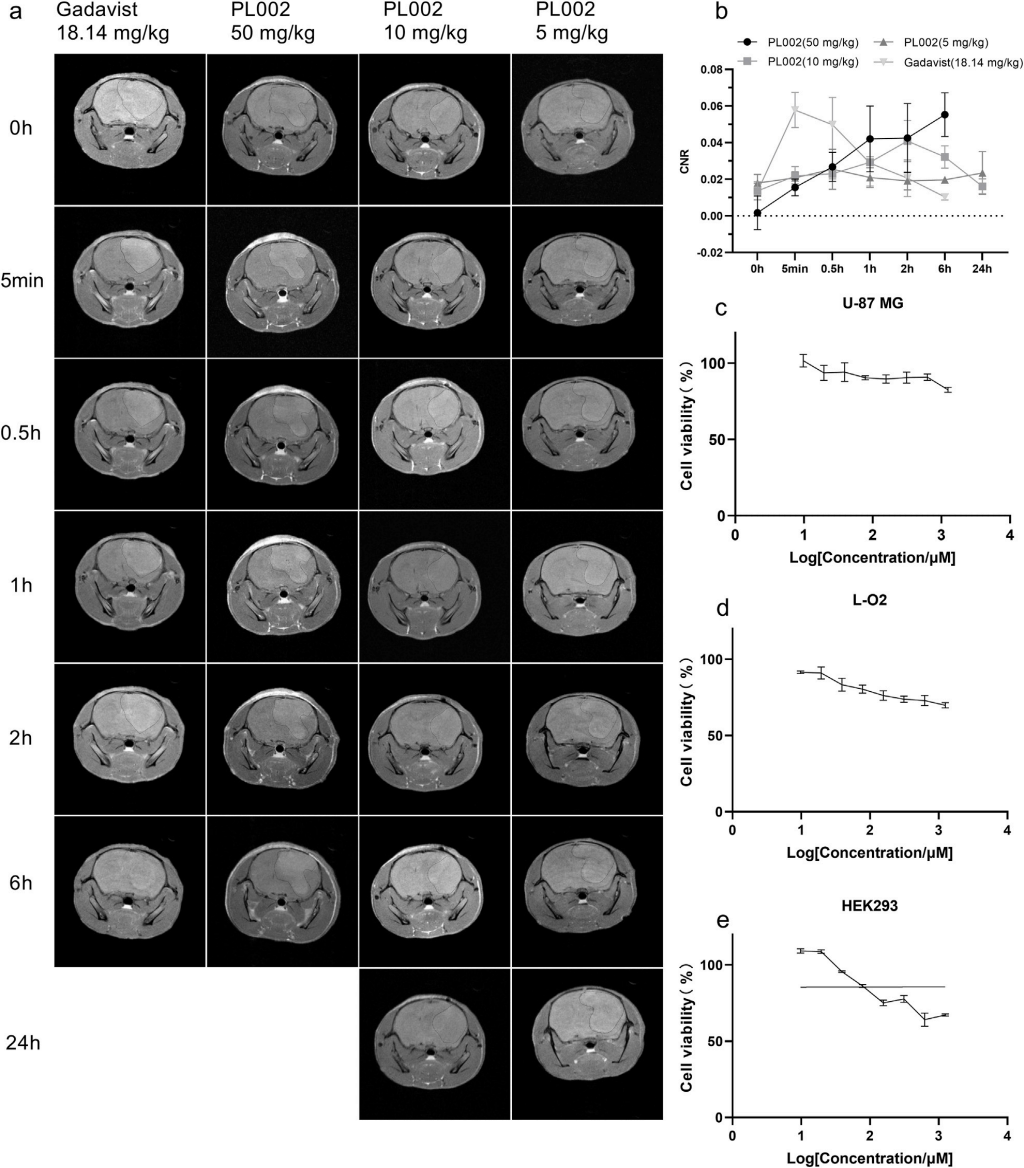
MR Imaging *in vivo*. **(a)** MR Imaging of PL002 and Gadavist^®^ groups (PL002: ① 50 mg/kg, 30 uM Gd3+&ICG/kg; ② 10 mg/kg, 6 uM Gd3+&ICG/kg; ③ 5 mg/kg, 3 uM Gd3+&ICG/kg; GadavistR: ④ 18.14 mg/kg, 30 uM Gd3+/kg). **(b)** Contrast result of MR Imaging. **(c–e)** represent the proliferation activity results of PL002 evaluated by the CCK-8 method in U-87 MG, L-O2, and HEK293 cells, respectively.

Before conducting *in vivo* imaging, the potential toxicity of PL002 on U-87 MG, L-O2, and HEK293 cell lines was assessed using the CCK-8 assay. They are used to assess the compound’s potential toxicity *in vitro*, serving as a reference for its use in glioblastoma multiforme (GBM) surgery. The cells were treated with PL002 at various concentrations (ranging from 0 to 1250 μM) for a period of 24 h, followed by a 45-min incubation with a 10% CCK-8 solution to assess the compound’s impact on cell growth. Figure 3 illustrates that PL002 does not exhibit cytotoxicity to U-87 MG, L-O2, and HEK293 cells at the tested concentrations (up to 1250 μM). These findings suggest that PL002 is biocompatible and has a low level of toxicity.

In comparative imaging experiments using a glioblastoma model, Gadavist^®^ was used as a positive control against PL002. As illustrated in Figure 3, T1-weighted turbo spin echo (TSE) enhanced scans revealed that tumors in the models appeared as circular or quasi-circular masses. In larger tumors, signs of necrosis and bleeding could be observed. The mass effect was evident as a shift of midline structures to the opposite side and compression, expansion, and displacement of the ipsilateral ventricle. After intravenous administration via the tail vein, clear images of brain tumor tissue were obtained. On T1-weighted MRI (T1WI), the lesion site appeared as a high-signal mass, with brain parenchymal signals lower than those in the tumor region. The tumor imaging effect was determined by comparing the CNR of the different treatment groups.

Post-administration, the CNR in the PL002 group increased, with the signal remaining relatively stable at 0.5 h. After 0.5 h, the CNR in the PL002 group showed a trend of increasing with higher doses. At 6 h, the high-dose PL002 group demonstrated superior imaging performance compared to other groups. Meanwhile, the low and medium dose groups of PL002 maintained a steady signal over 24 h. Additionally, the CNR of the Gadavist^®^ group increased rapidly post-administration, achieving optimal imaging effects at 5 min, and then gradually decreased. At 6 h, the signal of the Gadavist^®^ group was significantly lower than that of the PL002 groups (p-values for comparison with high-, medium-, and low-dose PL002 groups were 0.317, 0.009, and 0.033, respectively).

**FIGURE 4:**
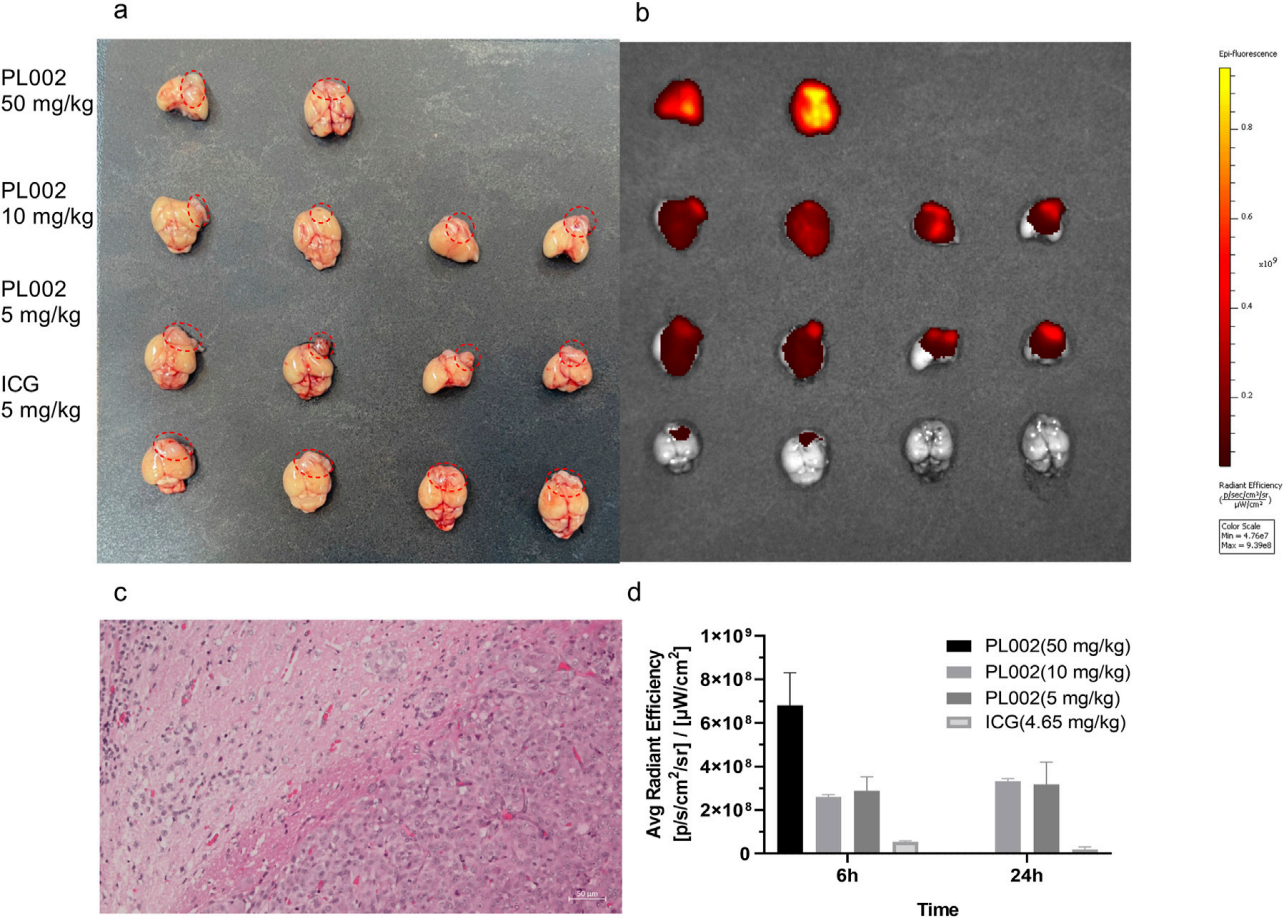
Fluorescent Imaging *in vivo*. **(a)** The brains in Fluorescent Imaging. **(b)** Fluorescent imaging result of PL002 and ICG groups. **(c)** HE staining figure. **(d)** Contrast result of Fluorescent Imaging (Due to the small sample size of only 2, no statistical analysis was performed.).

**FIGURE 5:**
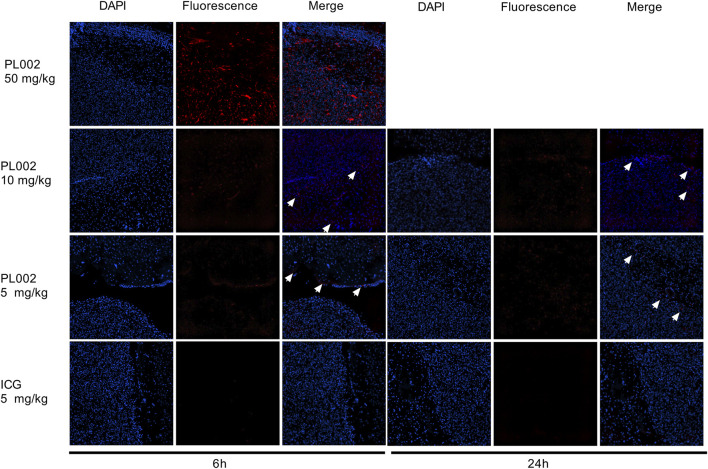
Fluorescence imaging of tumor microsections of PL002 and ICG groups in 6h and 24 h (200X; The white arrow indicates the location of the fluorescence).

After completion of MRI imaging, brain tissues were extracted from the PL002 and ICG groups at 6 h and 24 h post-administration, respectively. Fluorescence measurements were subsequently performed, with the results depicted in Figures 4, 5. ICG, serving as a fluorescent surgical marker, is capable of penetrating the blood-brain barrier and concentrating at tumor sites to facilitate targeted tumor imaging. However, fluorescence imaging revealed negligible fluorescence signals in the ICG group at both 6 and 24 h. In contrast, the PL002 group exhibited intense fluorescence within the tumor sites, with well-defined tumor margins and significantly higher mean fluorescence intensity compared to the ICG group. Moreover, comparison of tissue fluorescence and fluorescence intensity across different time points showed that, at 6 h, both normal tissues and blood vessels exhibited higher background fluorescence. In contrast, at 24 h, background fluorescence in normal tissues and blood vessels was reduced, resulting in more distinct tumor fluorescence margins and slightly higher mean fluorescence intensity compared to that at 6 h. Additionally, fluorescence microscopic sections revealed that only red fluorescence penetrated into the interior of the tumor without overlapping with the blue fluorescence of cell nuclei, with more red fluorescence aggregating at the tumor margins and in microvessels. These findings indicate that PL002 retains the passive targeting properties of ICG to pathological tissues, effectively crossing the blood-brain barrier, distributing around the tumor, and penetrating into the tumor interior along tumor vessels, thereby accumulating at the tumor site for an extended period.

### 3.3 Pharmacokinetics studies

**FIGURE 6:**
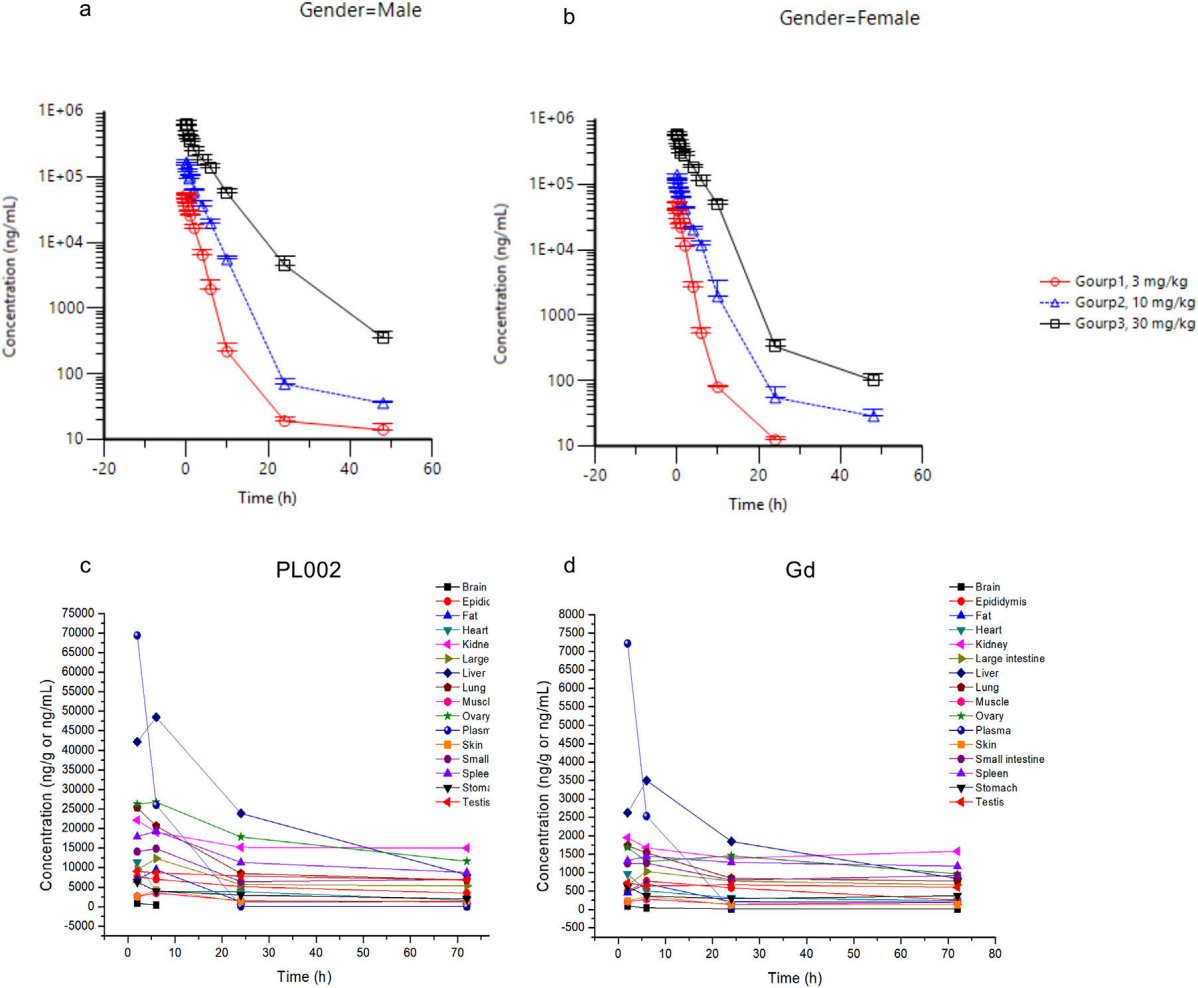
The pharmacokinetic profile and tissue distribution of PL002 *in vivo*. **(a)** The average plasma concentration in male animals of each dosage group; **(b)** The average plasma concentration in female animals of each dosage group; **(c)** Concentration of PL002 in plasma and major tissues; **(d)** Concentration of gadolinium in plasma and major tissues [In Figures **(a, b)**, plasma sampling time points are: pre-dose, 4 min, 10, 15, 30, and 45 min, and 1, 2, 4, 6, 10, 24, and 48 h post-administration; in Figures **(c, d)**, tissue sampling time points are: 2, 6, 24, and 72 h after dosing.].

Sprague-Dawley (SD) rats were used to evaluate the pharmacokinetic profile and tissue distribution of PL002. After a single intravenous injection of PL002 at dosages of 3 mg/kg, 10 mg/kg, and 30 mg/kg, the plasma concentration of PL002 peaked immediately following the infusion (at 4 min). Subsequently, it rapidly transitioned into the elimination phase, with an average terminal half-life of elimination ranging from 2.51 to 4.87 h (Figure 6).

Following the intravenous administration of PL002 at a dose of 10 mg/kg to SD rats, the compound demonstrated a degree of exposure across all tissues, indicating a widespread distribution. The findings revealed that PL002 was predominantly accumulated in tissues with abundant blood supply, such as the liver, kidneys, spleen, and lungs, as well as in the ovaries, small intestine, and testes. The distribution pattern of gadolinium within PL002 mirrored that of the compound itself, with a significant concentration observed in the liver, kidneys, spleen, lungs, ovaries, large intestine, and small intestine (Figure 6). This suggests that, post intravenous infusion, the dual-modality contrast agent predominantly exists in tissues and plasma in the form of the parent drug.

### 3.4 Safety study *in vivo*

Drawing from the pharmacokinetic studies mentioned, PL002 is characterized by a slower metabolic rate and prolonged retention in the body, which could potentially impose a significant physiological burden on subjects. To assess the safety of PL002, acute toxicity studies were conducted using SD rats following a single intravenous dose. The control group was administered a 5% glucose solution, whereas the experimental groups received PL002 at dosages of 100, 300, and 1,000 mg/kg. No adverse reactions were observed in the low-dose group rats; in the medium-dose group, 3 rats died within 30 min to 1 day after administration, and in the high-dose group, 10 rats died, suspected to be due to acute renal failure caused by PL002. Except for the low-dose group, animals exhibited reduced spontaneous activity, weight loss, and decreased appetite after administration, with more severe symptoms in the high-dose group. However, these conditions were reversible, and by the end of the 2-week observation period, the surviving rats had returned to normal, with body weight and food intake comparable to the control group. Gross anatomical observations revealed that the skin, adrenal glands, kidneys, testes, epididymis, ovaries and fallopian tubes, uterus, pancreas, lymph nodes (mesenteric and mandibular), and lungs appeared green in color. Histopathological examination showed varying degrees of cortical tubular necrosis in the kidneys, and minor injuries were also detected in the liver, heart, spleen, and lungs; the remaining discolored organs showed no abnormal findings under the microscope.

In summary, the acute toxicity of the test product led to significant mortality and adverse effects in the high-dose group. Conversely, the low-dose group did not show any unusual reactions. This indicates that while PL002 carries potential safety hazards at higher doses, it is deemed safe when administered at doses below 100 mg/kg.

## 4 Discussion

According to the 2022 Brain Glioma Diagnosis and Treatment Guidelines, the primary treatment for GBM involves surgical resection, complemented by radiotherapy and chemotherapy. The surgical objective is to safely remove the tumor to the greatest extent possible, with magnetic resonance imaging (MRI) playing a pivotal role throughout the procedure. Gadavist^®^ is a novel and potent MRI contrast agent approved for enhancing the visibility of various body parts, including the brain, spinal cord, blood vessels, liver, and kidneys (Szomolanyi et al., 2019; Knobloch et al., 2020; Accessdata, 2020). According to the guidelines, the optimal imaging results with Gadavist^®^ of MRI can be observed approximately 5 min post-injection, with tissue enhancement typically lasting until 45 min post-injection, which is suitable for preoperative imaging. Considering that glioblastoma surgery often lasts 4–5 h, if the surgeon needs to confirm the extent of resection during or after surgery, the patient would require another injection, imposing a significant physiological burden. Years of clinical research and post-marketing surveillance data have shown that Gadavist^®^ has a relatively high safety profile (Forsting and Palkowitsch, 2010; Endrikat et al., 2024), but there is still a low probability of mild to moderate adverse reactions occurring after administration, such as headache, nausea, injection site reactions, taste disturbances, and a sensation of warmth. Severe adverse reactions include cardiac arrest, respiratory arrest, and anaphylactic shock, which may be related to the distribution of gadolinium ions or the stability of the drug itself.

PL002 can immediately enhance MRI imaging after injection at a relatively low dose, with the imaging effect gradually increasing within 6 h post-injection, and the tumor imaging boundaries remaining clear. Combined with its pharmacokinetic profile, PL002 has a half-life exceeding 2 h and prolonged *in vivo* circulation time, ensuring stable brain levels for up to 72 h. This stability fully supports pre-, intra-, and postoperative MRI needs for GBM surgery. In comparison, Gadavist^®^ shows a signal decline starting at 5 min post-injection, with significantly lower MRI signals in tumor regions at 6 h post-injection compared to PL002. The tumor boundaries become blurred, and a single dose may not meet the imaging needs for the entire surgical procedure. Moreover, glioblastoma originates from glial cells and is characterized by rapidly proliferating tumor cells that infiltrate diffusely along white matter fibers and perivascular spaces. This leads to ill-defined tumor margins and incomplete resection. Glioblastoma is often accompanied by cerebral edema, which not only increases intracranial pressure but also obscures the actual tumor boundaries, complicating diagnosis and treatment. Gadavist^®^ may have limitations in distinguishing tumor tissue from cerebral edema/infiltrative margins, as its short imaging window and mild enhancement signals in both cerebral edema and infiltrative margins reduce contrast and affect the accuracy of clinical diagnosis. However, PL002s prolonged retention and stable imaging within the tumor, coupled with the gradual decrease in drug concentration in normal tissues and edematous regions outside the tumor, enhance the sensitivity and specificity of PL002 in MRI diagnosis.

In addition to MRI, PL002 contains an ICG fluorescent moiety that provides fluorescence-guided technology. Fluorescence-guided techniques are also recommended as an adjunct in glioblastoma surgery. ICG is one of the commonly used clinical fluorophores, but its application in low-grade gliomas is limited. The accumulation of ICG in GBM and the delineation of brain boundaries are primarily attributed to the disrupted blood-brain barrier (BBB) and preferential accumulation in areas with enhanced vascular permeability due to defective vascular architecture, impaired lymphatic drainage, and increased permeability mediators (Ergin et al., 2012; Maeda et al., 2000). Clinical research results show that among four cases of brain gliomas assisted by ICG-guided surgery, the fluorescence positivity rate was 50%. In the two failed cases, ICG was administered 24 h before surgery (Bianconi et al., 2023). The long time interval between drug administration and surgery may have led to the clearance of the drug retained in the tumor. Moreover, ICG itself has a rapid metabolism, and the relatively less disrupted BBB in patients with low-grade gliomas results in insufficient accumulation of ICG in brain tumors (Watson et al., 2018). Coupled with the extracellular accumulation and uneven distribution of ICG within tumor tissues, it may not accurately delineate tumor boundaries (Onda et al., 2016). This was also demonstrated in the fluorescence imaging at 6 and 24 h post-administration of ICG in this study. In contrast, PL002 crosses the blood-brain barrier in a manner similar to ICG and retains the advantage of passive accumulation in the tumor region. At 6 and 24 h post-injection, the fluorescent boundaries of the tumor region in the PL002 group remained stable and clearly delineated the tumor area. The slower metabolism of PL002 resulted in brighter and clearer fluorescent boundaries in the tumor region at 24 h, with higher average fluorescence intensity in the tumor area.

Finally, safety is an inevitable consideration in clinical applications. In imaging and pharmacokinetic studies, PL002 has been found to be highly stable, with its tissue distribution closely matching that of gadolinium ions. This indicates that PL002 primarily exists in its original form within the body after administration. Combined with acute toxicity studies, intravenous administration of PL002 at doses up to 100 mg/kg revealed no abnormalities, suggesting that there are likely no safety concerns in the short term. Moreover, compared to Gadavist^®^, PL002 has a longer half-life. According to tissue distribution data, after the same half-life period, the residual amount of gadolinium in tissues from PL002 may be lower than that from Gadavist^®^. The drug clearance from the body may be more extensive for PL002, which is advantageous for its long-term safety. Additionally, PL002 demonstrates good magnetic resonance imaging (MRI) and excellent fluorescence imaging at dosages of 10–50 mg/kg (0.006–0.03 mmol Gd/kg). The content of gadolinium in PL002 is less than 10%, which means that PL002 can be used at relatively lower doses in clinical applications, with correspondingly lower doses of gadolinium. To some extent, this can mitigate the safety concerns related to gadolinium deposition.

## 5 Conclusion

In summary, PL002 combines the advantages of MRI and fluorescence imaging while retaining the passive tumor targeting and strong imaging signal of ICG. Its extended half-life addresses the short imaging window issues associated with GBCAs and ICG, offering high tissue resolution, strong penetration, and high sensitivity. This ensures effective imaging while also having a broad therapeutic dose range. All of these features suggest that PL002 has promising application values and development prospects in the evaluation of resection margins before surgery, real-time intraoperative navigation, and postoperative confirmation of the extent of resection.

## Data Availability

The original contributions presented in the study are included in the article/Supplementary Material, further inquiries can be directed to the corresponding authors.

## References

[B1] Accessdata (2020). Gadavist product label. Available online at: https://www.accessdata.fda.gov/drugsatfda_docs/label/2011/201277s000lbl.pdf (Accessed october 6, 2020).

[B2] Acerbi F. Vetrano I. G. Sattin T. de Laurentis C. Bosio L. Rossini Z. (2018). The role of indocyanine green videoangiography with FLOW 800 analysis for the surgical management of central nervous system tumors: an update. Neurosurg. Focus 44 (6), E6. PMID: 29852759. 10.3171/2018.3.FOCUS1862 29852759

[B3] Bianconi A. Bonada M. Zeppa P. Colonna S. Tartara F. Melcarne A. (2023). How reliable is fluorescence-guided surgery in low-grade gliomas? A systematic review concerning different fluorophores. Cancers (Basel) 15 (16), 4130. PMID: 37627158; PMCID: PMC10452554. 10.3390/cancers15164130 37627158 PMC10452554

[B4] Bonosi L. Marrone S. Benigno U. E. Buscemi F. Musso S. Porzio M. (2023). Maximal safe resection in glioblastoma surgery: a systematic review of advanced intraoperative image-guided techniques. Brain Sci. 13 (2), 216. PMID: 36831759; PMCID: PMC9954589. 10.3390/brainsci13020216 36831759 PMC9954589

[B5] Endrikat J. Gutberlet M. Hoffmann K. T. Schöckel L. Bhatti A. Harz C. (2024). Clinical safety of gadobutrol: review of over 25 Years of use exceeding 100 million administrations. Invest. Radiol. 59 (9), 605–613. Epub 2024 Mar 1. PMID: 38426761. 10.1097/RLI.0000000000001072 38426761

[B6] Ergin A. Wang M. Zhang J. Y. Bruce J. N. Fine R. L. Bigio I. J. (2012). The feasibility of real-time *in vivo* optical detection of blood-brain barrier disruption with indocyanine green. J. Neurooncol 106 (3), 551–560. Epub 2011 Oct 1. PMID: 21964696. 10.1007/s11060-011-0711-5 21964696

[B7] Eyüpoglu I. Y. Hore N. Savaskan N. E. Grummich P. Roessler K. Buchfelder M. (2012). Improving the extent of malignant glioma resection by dual intraoperative visualization approach. PLoS One 7 (9), e44885. Epub 2012 Sep 26. PMID: 23049761; PMCID: PMC3458892. 10.1371/journal.pone.0044885 23049761 PMC3458892

[B8] Forsting M. Palkowitsch P. (2010). Prevalence of acute adverse reactions to gadobutrol--a highly concentrated macrocyclic gadolinium chelate: review of 14,299 patients from observational trials. Eur. J. Radiol. 74 (3), e186–e192. Epub 2009 Jul 2. PMID: 19574008. 10.1016/j.ejrad.2009.06.005 19574008

[B9] Knobloch G. Frenzel T. Pietsch H. Jost G. (2020). Signal enhancement and enhancement kinetics of gadobutrol, gadoteridol, and gadoterate meglumine in various body regions: a comparative animal study. Invest. Radiol. 55 (6), 367–373. PMID: 31985602. 10.1097/RLI.0000000000000645 31985602

[B10] Krivosheya D. Rao G. Tummala S. Kumar V. Suki D. Bastos D. C. A. (2021). Impact of multi-modality monitoring using direct electrical stimulation to determine corticospinal tract shift and integrity in tumors using the intraoperative MRI. J. Neurol. Surg. A Cent. Eur. Neurosurg. 82 (4), 375–380. Epub 2019 Oct 28. PMID: 31659724. 10.1055/s-0039-1698383 31659724

[B11] Li P. Qian R. Niu C. Fu X. (2017). Impact of intraoperative MRI-guided resection on resection and survival in patient with gliomas: a meta-analysis. Curr. Med. Res. Opin. 33 (4), 621–630. Epub 2017 Feb 2. PMID: 28008781. 10.1080/03007995.2016.1275935 28008781

[B12] Maeda H. Wu J. Sawa T. Matsumura Y. Hori K. (2000). Tumor vascular permeability and the EPR effect in macromolecular therapeutics: a review. J. Control Release 65 (1-2), 271–284. PMID: 10699287. 10.1016/s0168-3659(99)00248-5 10699287

[B13] Mosteiro A. Di Somma A. Ramos P. R. Ferrés A. De Rosa A. González-Ortiz S. (2022). Is intraoperative ultrasound more efficient than magnetic resonance in neurosurgical oncology? An exploratory cost-effectiveness analysis. Front. Oncol. 12, 1016264. PMID: 36387079; PMCID: PMC9650059. 10.3389/fonc.2022.1016264 36387079 PMC9650059

[B14] Onda N. Kimura M. Yoshida T. Shibutani M. (2016). Preferential tumor cellular uptake and retention of indocyanine green for *in vivo* tumor imaging. Int. J. Cancer 139 (3), 673–682. Epub 2016 Apr 4. PMID: 27006261. 10.1002/ijc.30102 27006261

[B15] Ostrom Q. T. Price M. Neff C. Cioffi G. Waite K. A. Kruchko C. (2023). CBTRUS statistical Report: primary brain and other central nervous system tumors diagnosed in the United States in 2016—2020. Neuro-Oncology 25 (Suppl. ment_4), iv1–iv99. 10.1093/neuonc/noad149 37793125 PMC10550277

[B16] Qi Z. Yu H. Chen L. Qu Y. Zhang M. Qi G. (2024). Analysis and prediction of central nervous system tumor burden in China during 1990-2030. PLoS One 19 (4), e0300390. PMID: 38630737; PMCID: PMC11023588. 10.1371/journal.pone.0300390 38630737 PMC11023588

[B17] Schupper A. J. Rao M. Mohammadi N. Baron R. Lee J. Y. K. Acerbi F. (2021). Fluorescence-guided surgery: a review on timing and use in brain tumor surgery. Front. Neurol. 12, 682151. PMID: 34220688; PMCID: PMC8245059. 10.3389/fneur.2021.682151 34220688 PMC8245059

[B18] Schupper A. J. Roa J. A. Hadjipanayis C. G. (2022). Contemporary intraoperative visualization for GBM with use of exoscope, 5-ALA fluorescence-guided surgery and tractography. Neurosurg. Focus Video 6, V5. 10.3171/2021.10.focvid21174 PMC9555356 36284587

[B19] Staartjes V. E. Togni-Pogliorini A. Stumpo V. Serra C. Regli L. (2021). Impact of intraoperative magnetic resonance imaging on gross total resection, extent of resection, and residual tumor volume in pituitary surgery: systematic review and meta-analysis. Pituitary 24 (4), 644–656. Epub 2021 May 4. PMID: 33945115; PMCID: PMC8270798. 10.1007/s11102-021-01147-2 33945115 PMC8270798

[B20] Szomolanyi P. Rohrer M. Frenzel T. Noebauer-Huhmann I. M. Jost G. Endrikat J. (2019). Comparison of the relaxivities of macrocyclic gadolinium-based contrast agents in human plasma at 1.5, 3, and 7 T, and blood at 3 T. Invest. Radiol. 54 (9), 559–564. PMID: 31124800; PMCID: PMC6738537. 10.1097/RLI.0000000000000577 31124800 PMC6738537

[B21] Tsugu A. Ishizaka H. Mizokami Y. Osada T. Baba T. Yoshiyama M. (2011). Impact of the combination of 5-aminolevulinic acid-induced fluorescence with intraoperative magnetic resonance imaging-guided surgery for glioma. World Neurosurg. 76 (1-2), 120–127. PMID: 21839963. 10.1016/j.wneu.2011.02.005 21839963

[B22] Watson J. R. Martirosyan N. Lemole G. M. Trouard T. P. Romanowski M. (2018). Intraoperative brain tumor resection with indocyanine green using augmented microscopy. J. Biomed. Opt. 23(9):1–4. 10.1117/1.jbo.23.9.090501 PMC6170140 30251491

[B23] Wirtz C. R. Albert F. K. Schwaderer M. Heuer C. Staubert A. Tronnier V. M. (2000). The benefit of neuronavigation for neurosurgery analyzed by its impact on glioblastoma surgery. Neurol. Res. 22 (4), 354–360. PMID: 10874684. 10.1080/01616412.2000.11740684 10874684

