# Supplementary material for: A preclinical study of a novel dual-modality contrast agent in rodent models
Source: Front Bioeng Biotechnol. 2025 Mar 20;13:1557772. doi: 10.3389/fbioe.2025.1557772 (PMC11966174; doi:10.3389/fbioe.2025.1557772)

天津药明康德新药开发有限公司
WuXi AppTec(Tianjin) Co., Ltd.
Address: No. 6 Building, 168 Nanhai Road 10th Avenue TEDA, Tianjin, P.R. China
Email：xing_jianguang@wuxiapptec.com

WuXi AppTec (Tianjin) Co., Ltd.

FL00001-1385 结构确证报告

FL00001-1385 Structure Confirmation Report

June 24, 2022

项目负责人 Project leader：远莹莹
项目试验人 Project tester：邢建广、时嘉敏、高祉婧
实验日期 Test date：2022.05-2022.06

目录
Table of Contents

1 结果汇总 Summary of results 1
2 紫外光谱 Ultraviolet spectrum.. 1
3 红外光谱 Infrared spectrum 2
4 高分辨质谱 High resolution mass spectrometry 2

1 结果汇总 Summary of results

本报告对浙江普利药业有限公司提供的样品（Compound ID: PL002-00，Batch No：00-003052-41-P1，WuXi Sample ID: FL00001-1385），通过UV，IR，HRMS进行综合解析，分析检测结果与表1-1所示结构相符。

In this report, the sample provided by Zhejiang Puli Pharmaceutical Co., Ltd. (Compound ID: PL002-00, Batch No: 00-003052-41-P1, WuXi Sample ID: FL00001-1385) was comprehensively elucidated by UV, IR, HRMS. The analytical test results were consistent with the structure shown in Table 1-1.

Table 1-1. FL00001-1385 结构信息 FL00001-1385 Structural information

物质名称 Substance Name	化学结构/化学名称 Chemical Structure/Chemical Name
FL00001-1385 ($C_{81}H_{105}GdN_8O_{15}S_2$ /Exact Mass: 1651.6382)	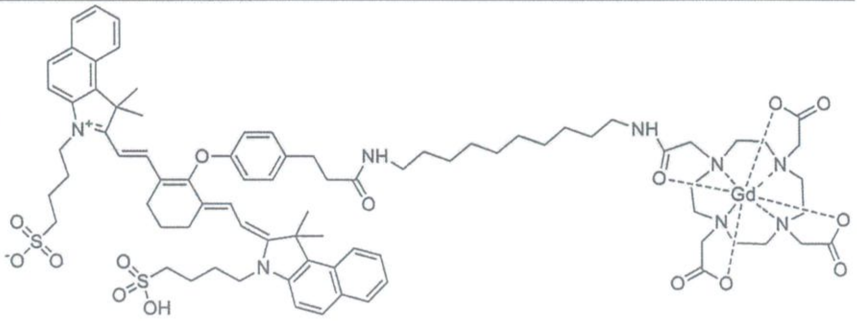 Name: gadolinium(III)2,2',2''-(10-(2-((10-(3-(4-(((E)-6-((E)-2-(1,1-dimethyl-3-(4-sulfobutyl)-1H-benzo[e]indol-2(3H)-ylidene)ethylidene)-2-((E)-2-(1,1-dimethyl-3-(4-sulfonatobutyl)-1H-benzo[e]indol-3-ium-2-yl)vinyl)cyclohex-1-en-1-yl)oxy)phenyl)propanamido)decyl)amino)-2-oxoethyl)-1,4,7,10-tetraazacyclododecane-1,4,7-triyl)triacetate

2 紫外光谱 Ultraviolet spectrum

Table 2-1 样品的紫外光谱吸收峰 UV absorption peak of sample

λ_{max}(nm)	Abs

216.5	0.248
805.0	0.915

最大吸收峰波长 λ_{max} = 216.5 nm 处为苯环引起的吸收带，表明结构中存在苯环，最大吸收峰波长 λ_{max} = 805.0 nm 处为三价 Gd 引起的吸收带，与表 1-1 中所示的 FL00001-1385 的结构式相符。

The maximum absorption peak wavelength λ_{max} = 216.5 nm was the absorption band caused by benzene ring, indicating that the presence of benzene ring in the structure; the maximum absorption peak wavelength λ_{max} = 805.0 nm was the absorption band caused by trivalent Gd, which was consistent with the structural formula of FL00001-1385 shown in Table 1-1.

3 红外光谱 Infrared spectrum

IR γ KBr max cm^{-1}: 2922.86、1620.75、1556.51、1506.89、1467.04、1400.51、1357.47、1231.58、1167.99、1140.22、1115.75、1039.05，1009.33、919.97、892.63。

红外光谱中 2922.86 cm^{-1} 为 C-H 伸缩振动吸收峰，1620.75 cm^{-1} 为酰胺和羧酸 C=O 伸缩振动吸收峰，1357.47 cm^{-1}、1115.75 cm^{-1} 为磺酸 SO_2 伸缩振动吸收峰，1039.05 cm^{-1} 为 C-N 伸缩振动吸收峰，与表 1-1 中所示的 FL00001-1385 的结构式相符。

In the infrared spectrum, 2922.86 cm^{-1} was the C-H stretching vibration absorption peak, 1620.75 cm^{-1} was the amide and carboxylic acid C=O stretching vibration absorption peak, 1357.47 cm^{-1}, 1115.75 cm^{-1} were the sulfonic acid SO_2 stretching vibration absorption peak, 1039.05 cm^{-1} was the C-N stretching vibration absorption peak. These were consistent with the structural formula of FL00001-1385 shown in Table 1-1.

4 高分辨质谱 High resolution mass spectrometry

高分辨质谱中捕获到准分子离子峰 *m/z* 1652.6564 $[M+H]^+$，826.8281 $[M+2H]^{2+}$，实测的精确分子量与预测分子式 $C_{81}H_{106}N_8O_{15}S_2Gd$ $[M+H]^+$的理论计算值 1652.6460 高度吻合，Diff = 6.3 PPM；与表 1-1 中 FL00001-1385 结构式相符。

选取 1652 作为母离子进行 MSMS 二级质谱检测，推测出主要的碎片离子可能的结构。

The quasi-molecular ion peak *m/z* 1652.6564 $[M+H]^+$, 826.8281 $[M+2H]^{2+}$ were captured in the high-resolution mass spectrum, the measured exact molecular weight was highly consistent with the theoretical calculated value of 1652.6460 for the predicted molecular formula $C_{81}H_{106}N_8O_{15}S_2Gd$ $[M+H]^+$, Diff = 6.3 PPM, which was consistent with the structural formula of FL00001-1385 shown in Table 1-1. Secondary mass spectrometry MSMS mode analysis was performed using *m/z* 1652 as the parent ion, and the possible structures of the main fragment ions were speculated.

Table 4-1. FL00001-1385 的二级质谱碎片离子及可能的碎片结构 FL00001-1385 Fragment ions and possible fragment structures in the secondary mass spectrum

碎片离子峰<br>Fragment ion peak	可能的碎片结构<br>possible fragment structure
330.1161	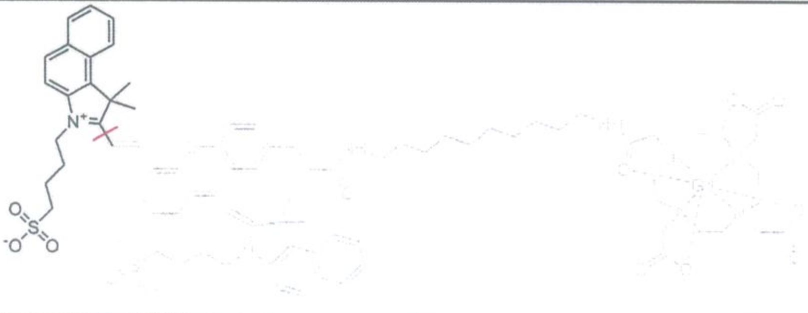
712.2827	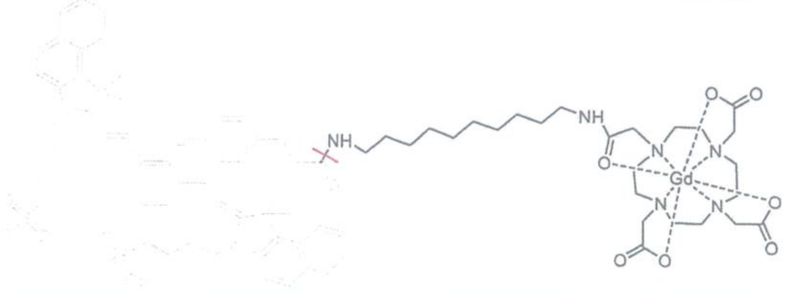

939.3781	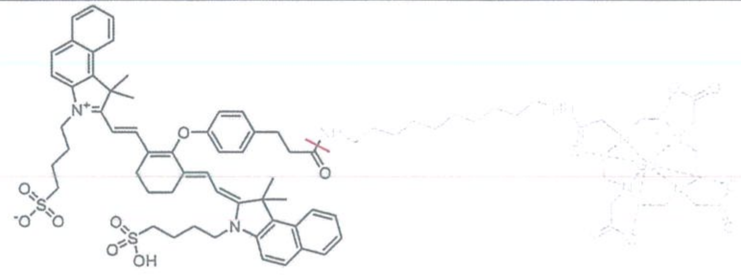
1321.5322	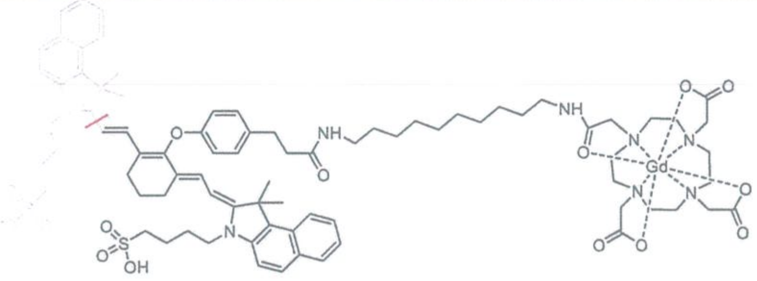

WuXi AppTec(Tianjin) Co., Ltd.Report

药明康德 WuXi AppTec

UV Report

06/15/2022 01:20:44 PM

[Measurement Properties]		Points:	4
Wavelength Range (nm.):	190.0 to 860.0	InterPolate:	Disabled
Scan Speed:	Medium	Average:	Disabled
Sampling Interval:	0.5		
Auto Sampling Interval:	Enabled	[Sample Preparation Properties]	
Scan Mode:	Auto	Weight:	
		Volume:	
[Instrument Properties]		Dilution:	
Instrument Type:	UV-2600 Series	Path Length:	
Measuring Mode:	Absorbance	Additional Information:	Sample: FL00001-1385 (190-860nm)
Slit Width:	2.0		
Accumulation time:	0.1 sec.		
Light Source Change Wavelength:	323.0 nm		
Detector Unit:	Direct		
S/R Exchange:	Normal		
Stair Correction:	OFF		
[Attachment Properties]			
Attachment:	6-Cell		
Number of cells:	1		
[Operation]			
Threshold:	~~0.0010000~~ 0.05		

ee WJR 13-Jun-2022

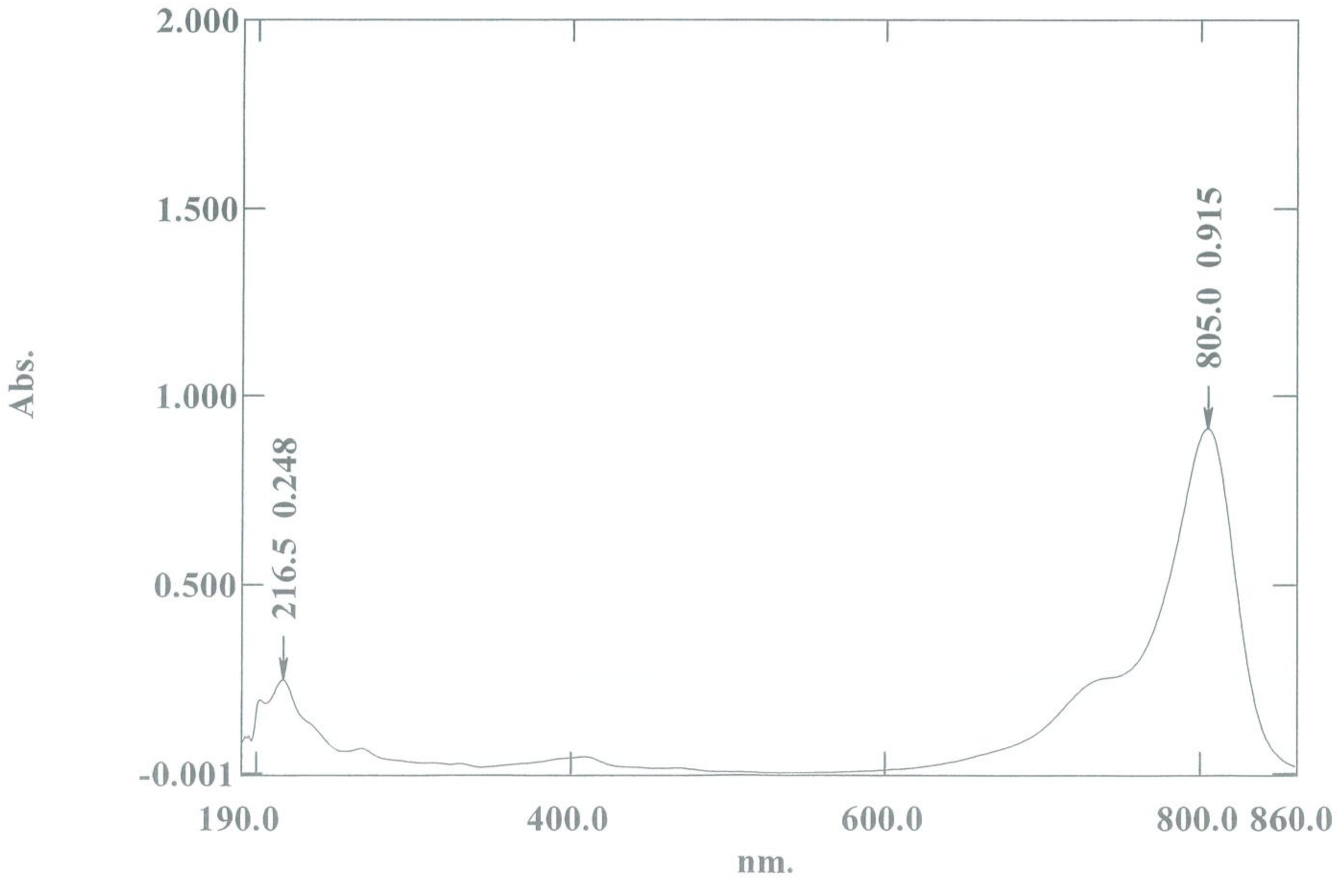

Junm Wei / 13-Jun-2022

Chengseng Zhang / 15-Jun-2022

IR Report

Results:

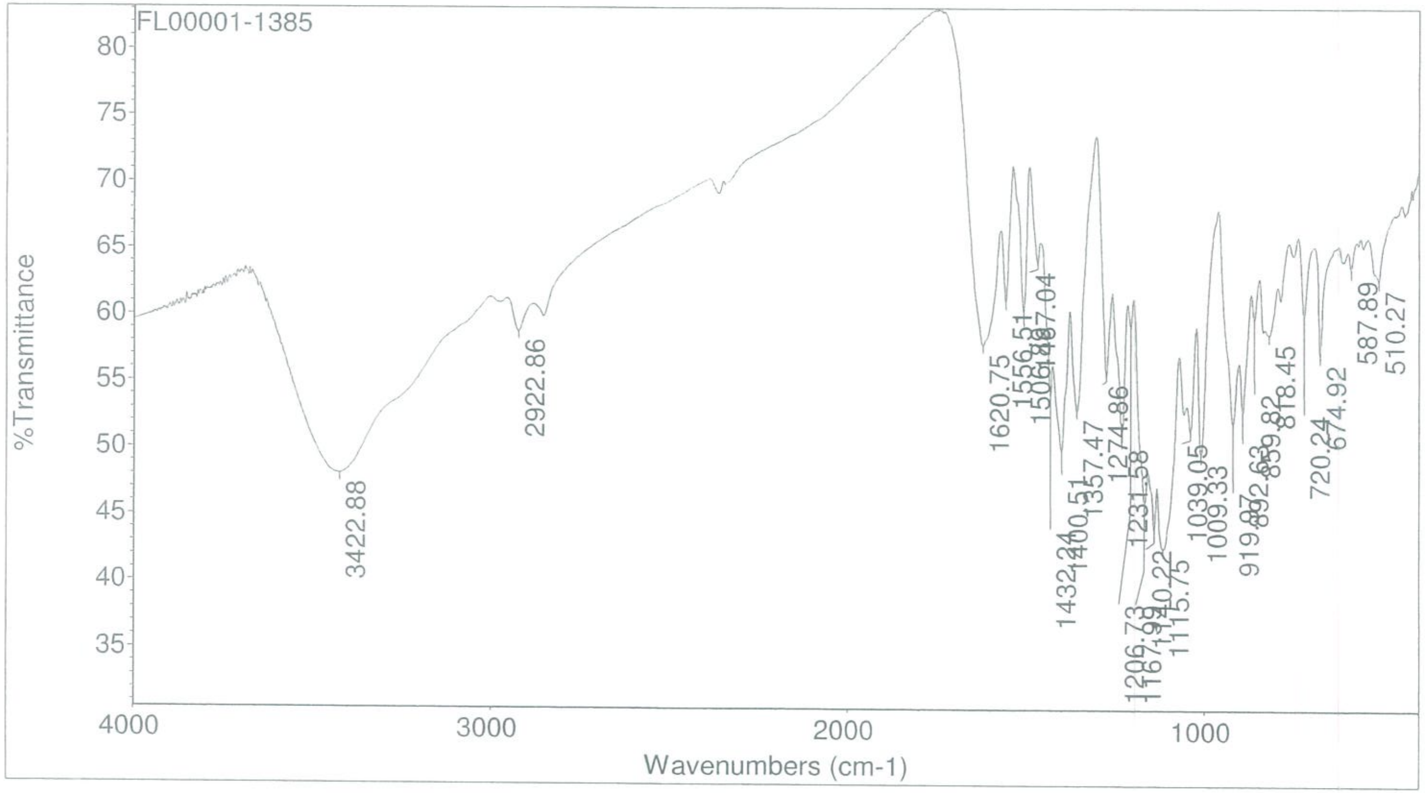

Junni Wen / 14-Jun-2022

Chengsong Zhang / 15-Jun-2022

天津药明康德新药开发有限公司
WuXi AppTec(Tianjin) Co., Ltd.

IR Report

Sample ID: FL00001-1385
Compound ID: PL002-00
Instrument: CAS-TJ-IR-01
Method: CAS-TJ-GTM-06.01
Detector: DTGS KBr
Beamsplitter: KBr
Source: IR
Number of sample scans: 32
Number of background scans: 32
Resolution: 4.000
Sample gain: 1.0
Optical velocity: 0.3165
Aperture: 80.00
Analysis time: Tue Jun 14 17:13:01 2022 (GMT+08:00)

Junm Wei / 14-Jun-2022
Chengsong Zhang / 15-Jun-2022

11:36:10
FL00001-1385_MS02

16-Jun-2022

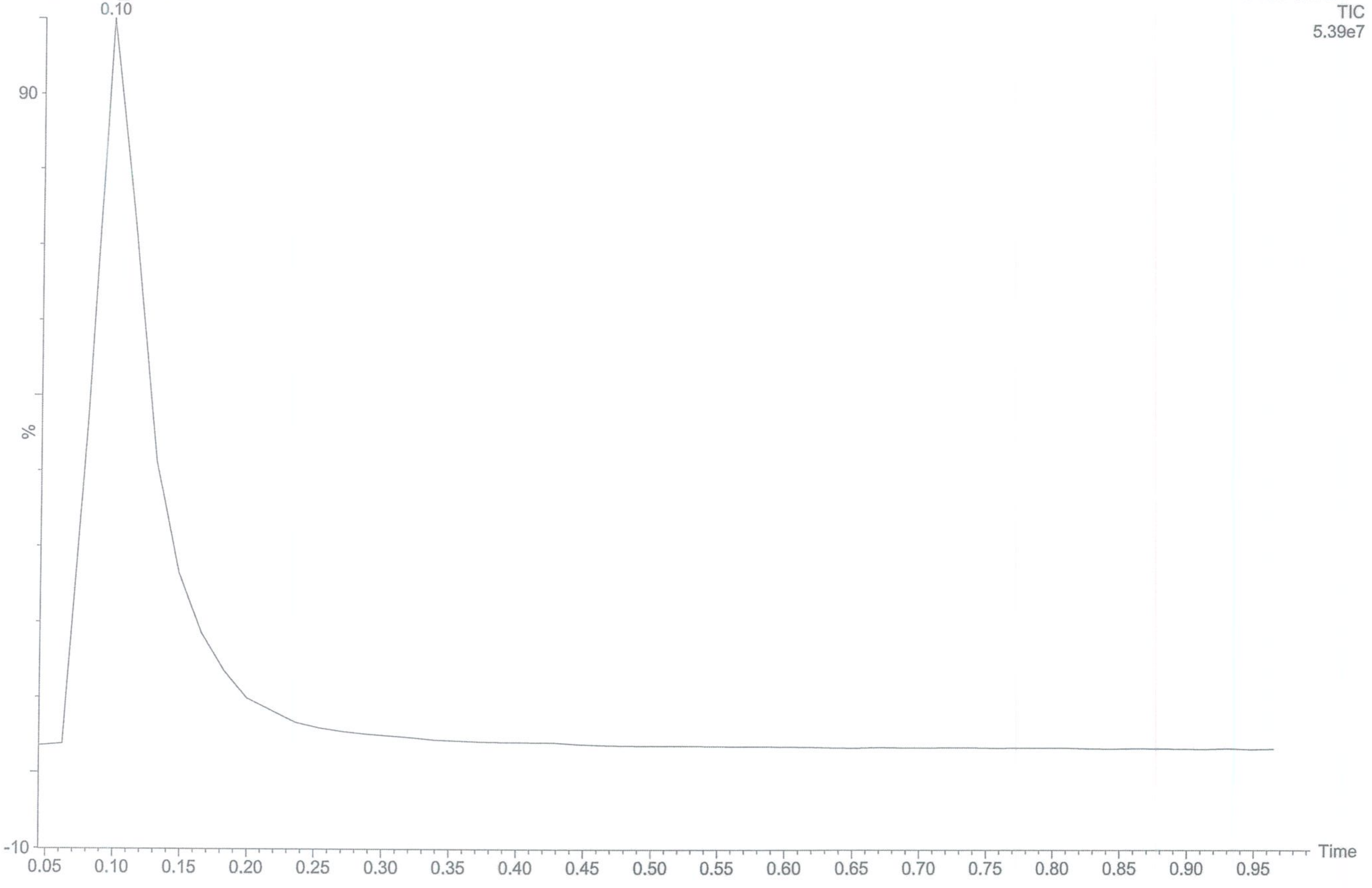

11:36:10
FL00001-1385_MS02 4 (0.097) Cm (3:9)

16-Jun-2022

1: TOF MS ES+
4.47e6

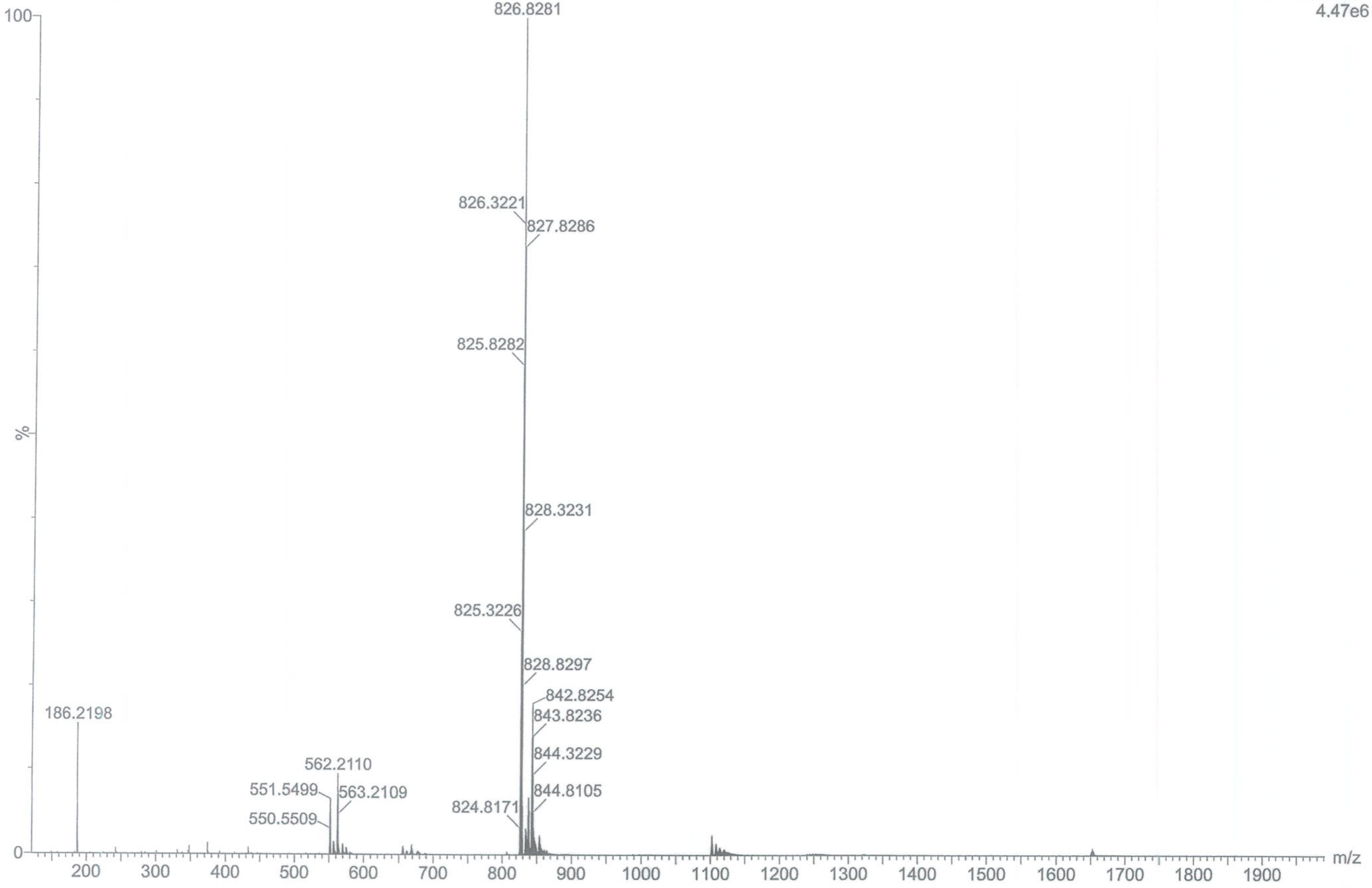

11:36:10 16-Jun-2022

FL00001-1385_MS02 4 (0.097) Cm (3:9) 1: TOF MS ES+ 3.99e4

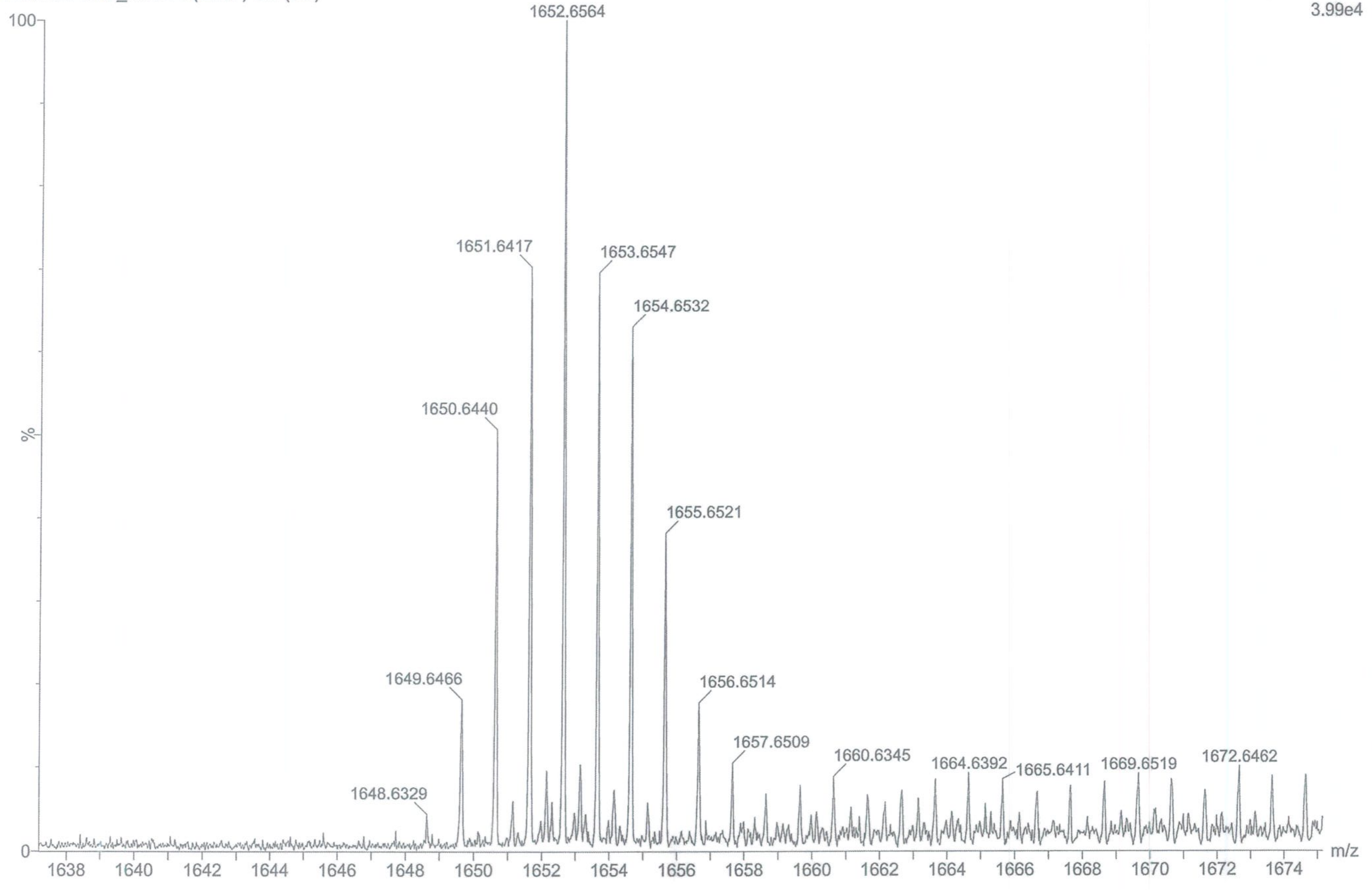

11:36:10
FL00001-1385_MS02 4 (0.097) Cm (3:9)

16-Jun-2022

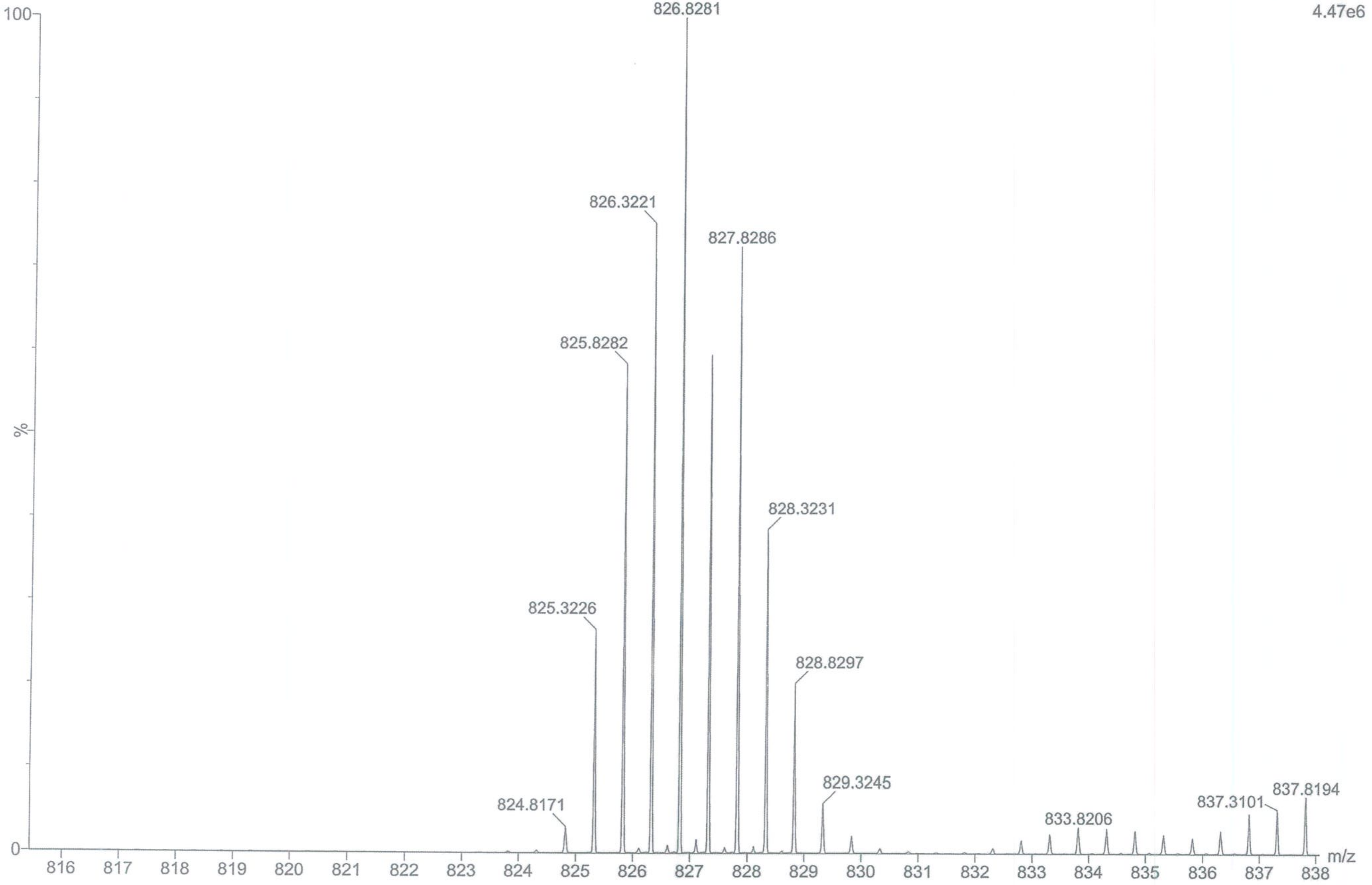

Elemental Composition Report

Single Mass Analysis

Tolerance = 100.0 PPM / DBE: min = -100.0, max = 100.0
Element prediction: Off

Monoisotopic Mass, Even Electron Ions
1 formula(e) evaluated with 1 results within limits (up to 50 closest results for each mass)
Elements Used:
C: 81-81 H: 106-106 N: 8-8 O: 15-15 S: 2-2 Gd: 1-1

11:36:10 16-Jun-2022
FL00001-1385_MS02 4 (0.097) Cm (3:9) 1: TOF MS ES+
3.99e+004

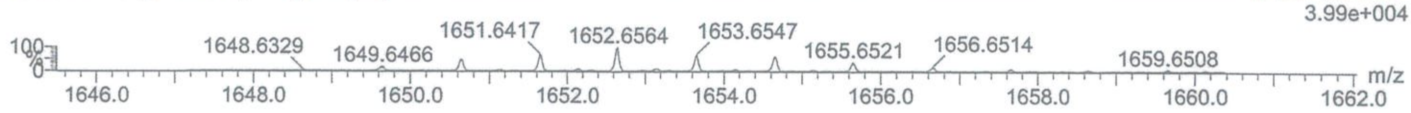

	Mass	Calc. Mass	mDa	PPM	DBE	Formula
Minimum:					-100.0	
Maximum:			5.0	100.0	100.0	
	1652.6564	1652.6460	10.4	6.3	33.5	C81 H106 N8 O15 S2 Gd

Zhijing Gao
27-Jun-2022

11:48:38

FL00001-1385_MSMS05

16-Jun-2022

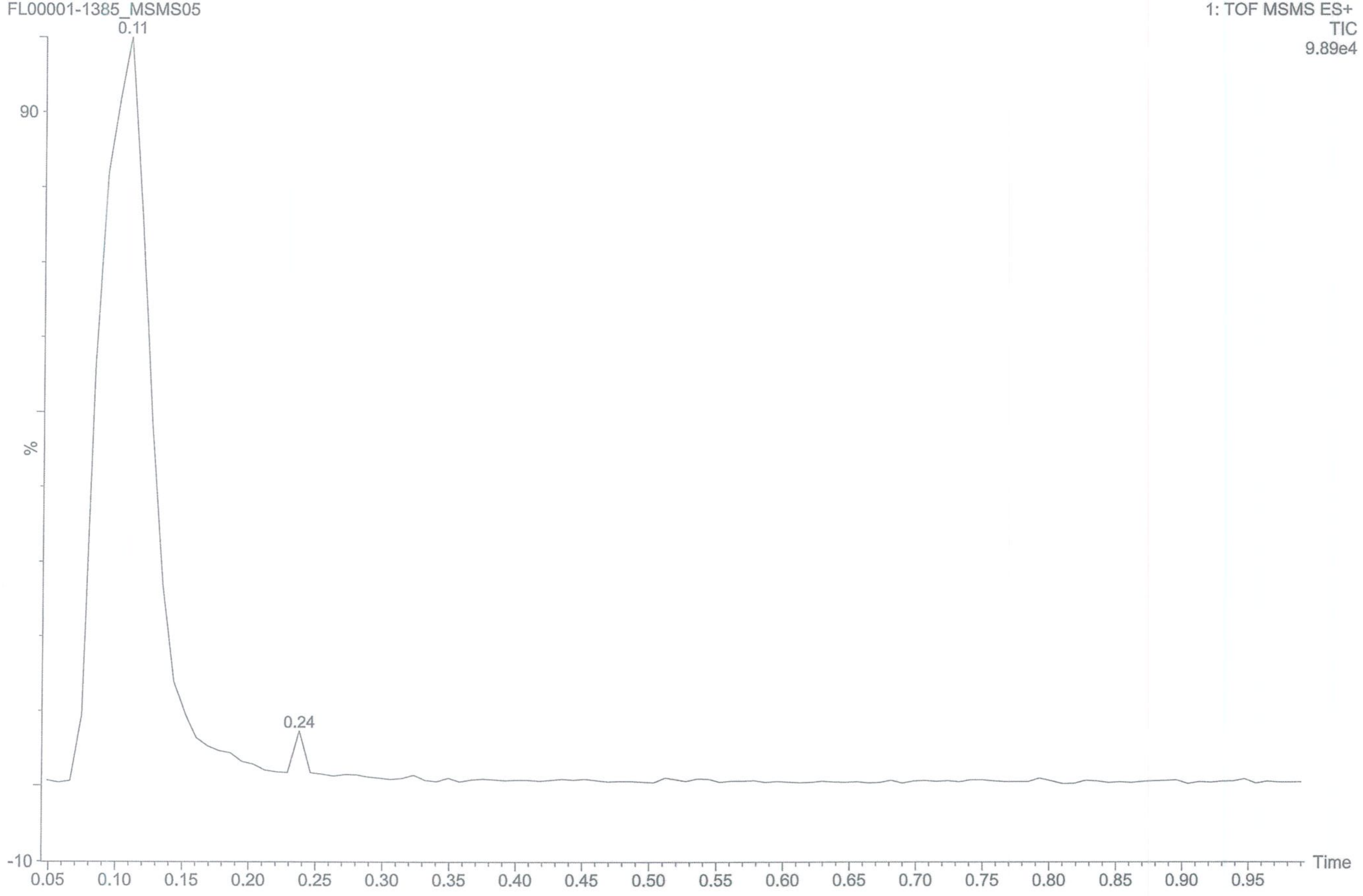

11:48:38
FL00001-1385_MSMS05 10 (0.109) Cm (7:14)

16-Jun-2022

1: TOF MSMS 1652.78ES+
2.56e4

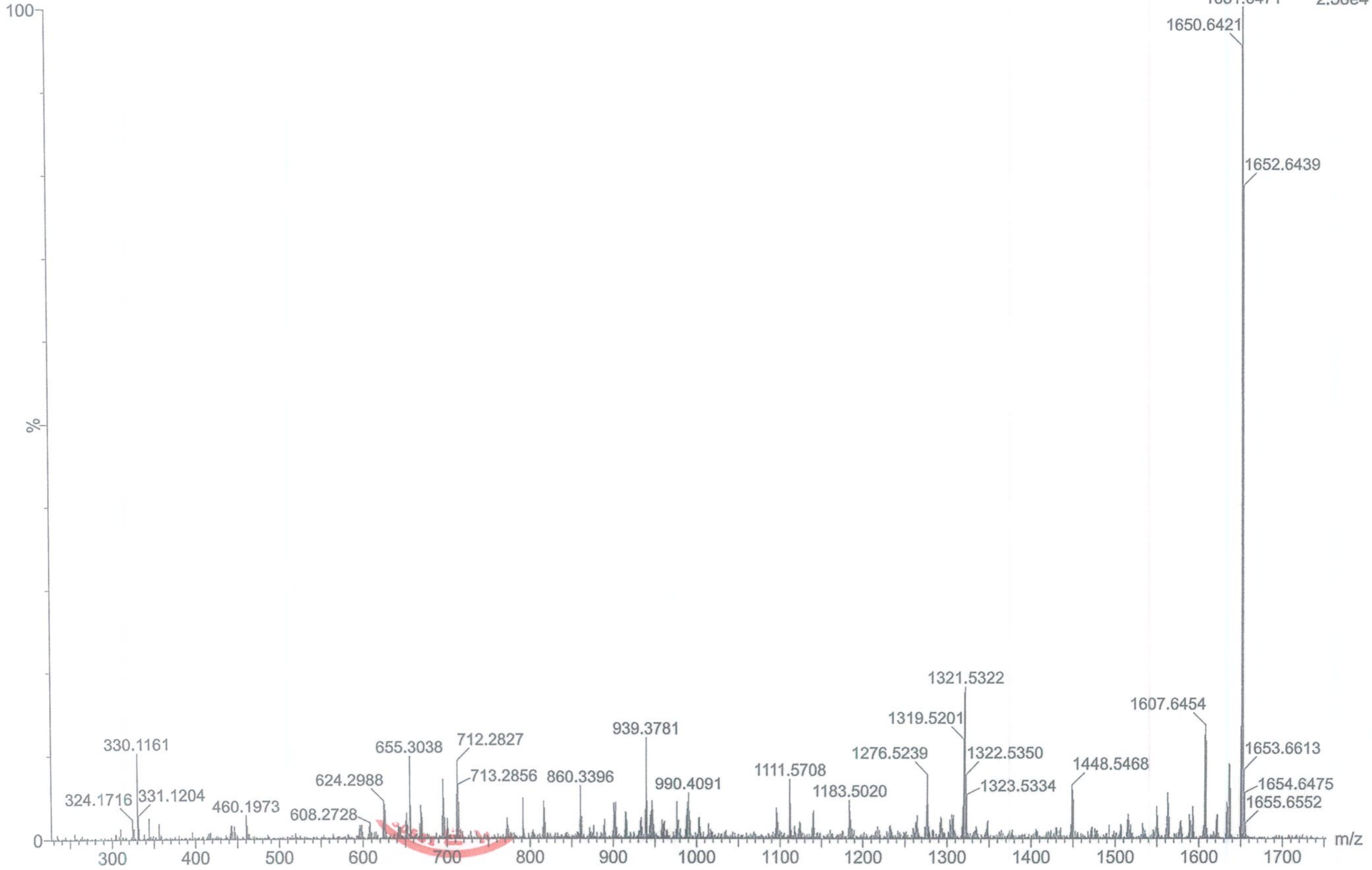

Supplement: Supplementary file 1 [file DataSheet1.pdf]
